# Network Path Convergence Shapes Low-Level Processing in the Visual Cortex

**DOI:** 10.3389/fnsys.2021.645709

**Published:** 2021-05-24

**Authors:** Bálint Varga, Bettina Soós, Balázs Jákli, Eszter Bálint, Zoltán Somogyvári, László Négyessy

**Affiliations:** ^1^Computational Neuroscience and Complex Systems Research Group, Department of Computational Sciences, Wigner Research Centre for Physics, Budapest, Hungary; ^2^János Szentágothai Doctoral School of Neurosciences, Semmelweis University, Budapest, Hungary; ^3^Faculty of Science and Engineering, University of Groningen, Groningen, Netherlands; ^4^Faculty of Information Technology and Bionics, Pázmány Péter Catholic University, Budapest, Hungary; ^5^Department of Anatomy, Histology and Embryology, Semmelweis University, Budapest, Hungary

**Keywords:** network resilience, oscillation, granger causality, anatomical hierarchy, hierarchical dynamics, hierarchical counterstream, network topology, shortest path

## Abstract

Hierarchical counterstream via feedforward and feedback interactions is a major organizing principle of the cerebral cortex. The counterstream, as a topological feature of the network of cortical areas, is captured by the convergence and divergence of paths through directed links. So defined, the convergence degree (CD) reveals the reciprocal nature of forward and backward connections, and also hierarchically relevant integrative properties of areas through their inward and outward connections. We asked if topology shapes large-scale cortical functioning by studying the role of CD in network resilience and Granger causal coupling in a model of hierarchical network dynamics. Our results indicate that topological synchronizability is highly vulnerable to attacking edges based on CD, while global network efficiency depends mostly on edge betweenness, a measure of the connectedness of a link. Furthermore, similar to anatomical hierarchy determined by the laminar distribution of connections, CD highly correlated with causal coupling in feedforward gamma, and feedback alpha-beta band synchronizations in a well-studied subnetwork, including low-level visual cortical areas. In contrast, causal coupling did not correlate with edge betweenness. Considering the entire network, the CD-based hierarchy correlated well with both the anatomical and functional hierarchy for low-level areas that are far apart in the hierarchy. Conversely, in a large part of the anatomical network where hierarchical distances are small between the areas, the correlations were not significant. These findings suggest that CD-based and functional hierarchies are interrelated in low-level processing in the visual cortex. Our results are consistent with the idea that the interplay of multiple hierarchical features forms the basis of flexible functional cortical interactions.

## 1. Introduction

Cognition emerges as the result of dynamics on the large-scale network of the cerebral cortex. Although large-scale cortical dynamics are rooted in the anatomical network formed by axonal connections between neuronal structures, the relationship of network architecture and function represented by coordinated network dynamics is far from clear. It is thought that modularity, core-periphery, and hierarchy are fundamental organizing principles of the anatomical network that constrain functioning. However, even the fundamental topological features exhibit large flexibility in temporally evolving functional networks (Bastos et al., 2015; Avena-Koenigsberger et al., 2018; Griffa and Van den Heuvel, 2018; Vezoli et al., 2021). A central question in understanding how network structure gives birth to the rich dynamics of cortical functioning is the role of topological features in identifying network paths of communication dynamics (Avena-Koenigsberger et al., 2018).

Shortest paths (the fewest number of links connecting any two nodes) play a fundamental role in exploring network characteristics (Newman, 2003); for an extensive review regarding brain networks see Avena-Koenigsberger et al. (2018). The brain network, constrained by its physical embedding, exhibits near-minimal path lengths, supporting the importance of shortest paths in large-scale neural functioning (Chklovskii et al., 2002; Chen et al., 2006). Accordingly, communication along shortest paths is highly efficient. However, it should be noted that shortest paths represent only a minor portion of possible communication paths and therefore are non-resilient, i.e., communication exclusively via shortest paths is prone to congestion, causing delays and loss of information. Consequently, multiple routing strategies have been suggested as functionally sensible alternatives to shortest paths (Avena-Koenigsberger et al., 2018). Besides network-wide communication, shortest paths play a significant role in determining the integrative and coordinative capacities at the level of network elements. It was shown e.g., that in a functional brain network the closeness centrality of an area (how easy it is to reach a node via the shortest paths) influences the path length of anatomical connections between active loci, resulting in a small but not negligible elongation (~10%) compared to the shortest path length (Csoma et al., 2017).

An important but often neglected property of the cortical network is that it is composed of directed axonal pathways. Only the anatomical network constructed by way of tract-tracing can represent the direction of neural connections, which provide further opportunities to identify functionally relevant topological features (Passingham et al., 2002; Kaiser, 2007; Markov et al., 2013a, 2014). Exploiting information in the distribution of directed edges and using edge betweenness centrality as a measure of connectedness (proportional to the number of shortest paths traversing an edge), an edge-based index called convergence degree (CD) was formulated, which uniquely characterizes the flow properties of networks (Négyessy et al., 2008; Bányai et al., 2011b). Considering all directed shortest paths traversing a given edge, the CD determines convergence or divergence by the difference in the number of source and target nodes, i.e., convergent edges convey information from a larger number of nodes to a smaller set, while divergent edges from a smaller to a larger set. In the cerebral cortex, CD revealed the mostly complementary divergent and convergent nature of reciprocal links and that this complementarity resulted in the formation of nearly symmetrical forward and backward subnetworks. Furthermore, the sum of the inward and outward convergence degrees of a node provided the node-centric representation of convergence/divergence, which is a useful measure of the coordinative function of cortical areas (Négyessy et al., 2008, 2012). Specifically, low-level areas including primary sensory cortices, serve as sources of information via their mostly divergent outputs and convergent inputs. On the contrary, high-level areas such as the prefrontal cortex receive mostly divergent inputs and provide convergent outputs, which make such regions information allocating structures, consistent with the role of controlling the flow of information in the cerebral cortex. In large-scale anatomical networks, in the same fashion as with the anatomical hierarchy, which is determined by the laminar distribution of projections, the areas' role changes gradually between source and allocating due to the combination of convergence/divergence properties in their inputs and outputs (Felleman and Van Essen, 1991; Markov et al., 2013a, 2014). Considering that the anatomical network represents cortical areas as single nodes and axonal bundles as links, these observations suggest that CD determines a fundamental, functionally relevant structural property of networks. More importantly, the dissociation of the reciprocal forward and backward subnetworks resemble very closely the counterstream hierarchical architecture of the cerebral cortex (Markov et al., 2013a; Vezoli et al., 2021). However, it remained to be seen to what extent CD determines cortical dynamics, most notably synchronization, where convergence and divergence are crucial network properties.

In the cerebral cortex, rhythmic synchronous oscillations are distinguishing neurophysiological indicators of different cognitive operations (Buzsáki and Draguhn, 2004; Wang, 2010). Amongst the rich pattern of oscillatory activity, theta, alpha-beta, and gamma band synchronizations play a pivotal role in cognitive functions. Remarkably, experimental evidence indicates that hierarchical cortical processing is characterized by a feedforward-related enhancement of gamma and theta band synchronization, and a feedback-related increase of alpha-beta band synchronization (Bastos et al., 2015, 2018, 2020; Lundqvist et al., 2020). Cortical computation is based on the interplay of synchronous oscillatory activities resulting in e.g., suppression, enhancement, and phase locking (Buzsáki and Draguhn, 2004; Wang, 2010). What are the circuit mechanisms of hierarchical computation is less understood. Considering that middle and upper layers are the major targets of feedforward afferents, and feedback pathways are mostly associated to the deep layers, hierarchical processing is surmised to be the result of the interaction of these connectional activities across cortical laminar circuitries (Vezoli et al., 2021). Large-scale hierarchical cortical dynamics were recently studied by Mejias et al. (2016) using a laminar neuronal mass model in a weighted and directed anatomical network. The model faithfully reproduced different physiological phenomena of cortical interactions, both within and across the upper and lower layers, by using an experimentally measured coupling parameter determining anatomical hierarchical relationship, which is defined as the proportion of supragranular layer projecting neurons (Barone et al., 2000; Markov et al., 2014). Most notably, a specific, hierarchically dependent causal functional connection was reproduced between the areas in gamma and alpha-beta band synchronization, as shown by electrophysiological observations (Bastos et al., 2015). These observations were in great agreement with the hierarchical counterstream organization of the large-scale anatomical network (Markov et al., 2013a; Vezoli et al., 2021).

The major goal of this study was to examine if the CD-based topological hierarchy, representing network-wide cortical convergence, plays a role in functional hierarchical interactions in the primate cerebral cortex. Our studies were focused on the large-scale anatomical network in the form of directed and either weighted or binary (considering only the existence of a connection) graphs (Sporns, 2007, 2010).

In the first part of the study, we aim to provide evidence that the CD is a sensitive measure of network integrity and synchronizability, i.e., a non-specific measure of a network's dynamic potential (Papo and Buldú, 2019). To this end, targeted and random edge removals were performed on an updated version of the binary, directed anatomical network representation of the macaque sensorimotor-visual cortex used in our previous investigations (Négyessy et al., 2006). Edges were removed on the basis of CD and edge betweenness (EB) values and the effects were compared to that obtained in similar experiments on randomized control networks. The lack of correlation between CD and EB (Négyessy et al., 2008) suggests that these two measures are independent. The effects of edge deletion were measured on network indices related to the efficiency of information transfer as well as robustness and synchronizability. Communication efficiency was studied via analyzing changes to the global topological distance, expressed as the average shortest path, and the diameter of the network (the longest of the shortest paths), while undergoing edge removal. Network synchronizability was studied by computing spectral graph metrics of the graph Laplacian (Chung, 1997; Boccaletti et al., 2006; Arenas et al., 2008; Chen et al., 2012). Specifically, the second smallest eigenvalue, λ_2_, which is known as algebraic connectivity (Fiedler, 1973), and is an important measure of graph robustness, i.e., its magnitude indicates connectedness. If λ_2_ is small, fewer edges are needed to be removed to disconnect the graph. In addition, the largest absolute eigenvalue (λ_*N*_), called the spectral radius was also computed. Synchronizability, as the stability of a synchronized state, is maximal in unweighted, fully random networks with uniform degree distribution, and depends strongly on λ_2_ (Nishikawa et al., 2003; Atay et al., 2006; Almendral and Díaz-Guilera, 2007; Chen et al., 2012). The higher the maximal eigenvalue, the easier the network reaches the synchronization regime. Also, a wider spread of the eigenvalues indicates an increased probability that the synchronous state will be unstable. The spread of the eigenvalues can be estimated by the ratio of the largest and smallest non-zero eigenvalues, called eigenratio (λ_2_/λ_*N*_). The closer the eigenratio is getting to 1 the more stable the synchronous state of the system becomes.

In the second part, the role of CD in hierarchical cortical interactions was studied by implementing the model of Mejias et al. (2016), simulating the oscillatory dynamics of a weighted and directed anatomical network of the macaque visual cortex. We used the only complete anatomical network consisting of 29 areas of the visual and prefrontal cortex available in primates (Markov et al., 2013b, 2014). It is a highly realistic structural representation that includes the direction and numerosity of axonal connections at the highest possible resolution (i.e., single axon or projection neuron). Large-scale hierarchical network dynamics were simulated by a bilaminar Wilson–Cowan model, suitable for studying the oscillatory characteristics of feedforward and feedback interactions (Mejias et al., 2016). First of all, we aimed to extend the use of CD to weighted networks, as this index has been defined and applied only for binary graphs before (Négyessy et al., 2008, 2012; Bányai et al., 2011b). To this end, a refined combination of existing methods was developed for finding shortest paths in weighted graphs, which favors robustness at the expense of a winner-take-all approach (Newman, 2001, 2005; Opsahl et al., 2010; Avena-Koenigsberger et al., 2018). The role of CD in cortical hierarchy was studied by way of correlations, both with an anatomy-based hierarchical index, and a frequency-dependent Granger causal coupling measure that was previously used in unraveling the functional hierarchy of the network (Bastos et al., 2015; Mejias et al., 2016). Furthermore, several hierarchically relevant features of the cerebral cortex are known that change gradually across the large-scale network of areas (Barone et al., 2000; Markov et al., 2014; Hilgetag et al., 2019; Hilgetag and Goulas, 2020; Wang, 2020; Vezoli et al., 2021). Therefore, to better understand the relationship between anatomical and topological features, we examined the role of anatomical hierarchical distances, i.e the pairwise difference in the levels that areas occupy in the anatomical hierarchy.

## 2. Materials and Methods

### 2.1. Networks

The network used for the edge removal experiments included areas of the macaque visual, somatosensory and motor cortices plus areas 46 and FEF of the prefrontal cortex. This network, referred to as the visuo-tactile network from now on, is the updated version of the one analyzed in our previous studies (Négyessy et al., 2006, 2008). The updated network consists of 44 nodes and 630 unweighted and directed edges. The connectivity and areal designation of the visuo-tactile network were updated with data from the core-nets.org database^1^ (Markov et al., 2014), complemented by extensive literature search (the connectivity matrix along with differences compared to the original network and the sources of information are summarized in the Binary_Visuotact_44x44.xlsx table included in the Supplementary Material).

Following Mejias et al. (2016) the dynamical simulations were based on a graph of 29 macaque visual areas, connected by 536 directed and weighted edges, which can be found in multiple datasets publicly available from core-nets.org (Markov et al., 2014); all data used were collected in the Weighted_Visual_29x29.xlsx table included in the Supplementary Material. This network comprises three types of edge information based on retrograde tracing experiments: (i) the physical distance of the projection, (ii) the anatomical strength of the projection (fraction of labeled projection neurons, FLN) used as network weights, and (iii) a hierarchical index of the connections (ratio of the supragranular labeled projection neurons, SLN) (Markov et al., 2014). Based on a single retrograde tracer injection in area *j*, the FLN and SLN for the directed connection from area *i* to area *j* can be written in equation forms as:

(1) $$\begin{array}{c} \text{FLN}\left(i,j\right)=\frac{\text{No. of labelled neurons in }i}{\text{Total no. of labelled neurons }},\\ \text{SLN}\left(i,j\right)=\frac{\text{No. of supragranular labelled neurons in }i}{\text{No. of supragranular }+\text{infragranular labelled neurons in }i}. \end{array}$$

Due to computational limitations, the final analyses, involving spectral Granger causal interactions, were primarily done in a selected 8 × 8 subgraph composed of areas V1, V2, V4, DP, 8l, 8m, TEO, and 7A, which were also the subject of previous studies (Bastos et al., 2015; Mejias et al., 2016).

### 2.2. Graph Metrics

For every pair of nodes *i* and *j* ∈ *V*, one can define the length of the *shortest path* between *i* and *j*, as the minimum number of edges one must traverse to reach *j* from *i* (*d*_*ij*_). The *diameter* of the cortical network is defined as the maximum of all shortest path lengths over all *i*-*j* pairs. If the network is disconnected (i.e., there exist *i* and *j* such that *j* cannot be reached from *i*), then the global diameter is taken to be the largest of the diameters computed for the individual components. The average shortest path length (ASP) of the network is the average of the length of all shortest paths for all *i*-*j* pairs:

(2) $$\begin{array}{l} \text{ASP}=\frac{\sum_{i=1}^{\left|V\right|}\sum_{j\neq i}^{\left|V\right|}d_{ij}}{|V|\left(|V|-1\right)}. \end{array}$$

The sum of the fractions of the number of shortest paths (*n*_*d*_*ij*__) for all node-pairs that traverse a given edge (*e*) is denoted as the *edge betweenness* centrality (EB):

(3) $$\begin{array}{l} \text{EB}\left(e\right)=\overset{\left|V\right|}{\underset{i=1}{\sum }}\overset{\left|V\right|}{\underset{i\neq j=1}{\sum }}\frac{n_{d_{ij}}\left(e\right)}{n_{d_{ij}}}. \end{array}$$

The *cluster index* (CI) of a given node quantifies how close the node and its neighbors are to being a clique (a complete subgraph where every node is connected to every other node in the clique) (Watts and Strogatz, 1998). The *global transitivity* was calculated as the cluster index of the whole network, which is the ratio of the triangles and connected triples in the graph:

(4) $$\begin{array}{l} \text{CI}=\frac{\text{No. of triangles in the network}}{\text{No. of triplets in the network}}\\ \;=\frac{\text{No. of connected triplets in the network }}{3\;\times \text{No. of triplets in the network}}. \end{array}$$

The direction of the edges was ignored in computing CI. A *strongly connected component* of a directed graph is a subgraph in which there is a directed path between any two vertices in both directions.

*Eigenvalues* characterizing robustness and synchronization related to the topological properties of the graph were derived from the graph Laplacian (Boccaletti et al., 2006). The second smallest (λ_2_) and the largest (λ_*N*_) eigenvalues were computed. Since the Laplacian is a positive semidefinite matrix with zero row sums, it always has the smallest eigenvalue zero with the corresponding eigenvector [1, 1, 1, 1…]^*T*^. The second smallest (and the largest) eigenvalue can be found by minimizing (maximizing) the eigenvalue equation, x^*T*^Lx, where x can be vectors perpendicular to [1, 1, 1, 1…]^*T*^ (Fiedler, 1973).

For the eigenratio, λ_2_/λ_*N*_ was used, which better indicated the dependence of the effect of edge removal on λ_2_ compared to λ_*N*_.

### 2.3. Convergence Degree (CD)

The degree of convergence and divergence was quantified by the analysis of all shortest paths using the notion of CD (Négyessy et al., 2008; Bányai et al., 2011b). CD is defined as the normalized difference between the number of input and target areas connected via a particular link of a directed graph, specifically

(5) $$\begin{array}{l} \text{CD}\left(i,j\right)=\frac{\left|In\left(i,j\right)|-|Out\left(i,j\right)\right|}{\left|In\left(i,j\right)\cup Out\left(i,j\right)\right|}, \end{array}$$

where *In*(*i, j*) denotes the set of nodes from where the shortest paths containing edge (*i, j*) emanate, while *Out*(*i, j*) denotes the set of nodes in which the shortest paths containing edge (*i, j*) terminate. |*In*(*i, j*)| denotes the cardinality of the set *In*(*i, j*). In the denominator the union is used because the input and output sets may have non-empty intersections, which are not considered here; see Bányai et al. (2011b) for a thorough treatise on the overlap. Positive CD value indicates a convergent connection since the input field of the connection contains more nodes than the output field, while the opposite is true for divergent edges characterized by negative CD values.

### 2.4. Node-Centric CD Representation

The positive and negative CD values of the incoming and outgoing edges were summed separately for each area, resulting in the node-centric CD representation of the network, called node-reduced, or nrCD (Négyessy et al., 2008). Thus, every area of the network is characterized by four numbers: the CD sum of incoming edges with positive and negative CD values, and the CD sum of outgoing edges with positive and negative CD values. The four equations are:

(6) $$\begin{array}{l} {\text{nrCD}}_{in}^{+}\left(i\right)=\frac{1}{n-1}\underset{j\in \Gamma_{in}\left(i\right)}{\sum }\Theta \left(\text{CD}\left(j,i\right)\right)\text{CD}\left(j,i\right), \end{array}$$

(7) $$\begin{array}{l} {\text{nrCD}}_{in}^{-}\left(i\right)=\frac{1}{n-1}\underset{j\in \Gamma_{in}\left(i\right)}{\sum }\Theta \left(-\text{CD}\left(j,i\right)\right)\text{CD}\left(j,i\right), \end{array}$$

(8) $$\begin{array}{l} {\text{nrCD}}_{out}^{+}\left(i\right)=\frac{1}{n-1}\underset{j\in \Gamma_{out}\left(i\right)}{\sum }\Theta \left(\text{CD}\left(i,j\right)\right)\text{CD}\left(i,j\right), \end{array}$$

(9) $$\begin{array}{l} {\text{nrCD}}_{out}^{-}\left(i\right)=\frac{1}{n-1}\underset{j\in \Gamma_{out}\left(i\right)}{\sum }\Theta \left(-\text{CD}\left(i,j\right)\right)\text{CD}\left(i,j\right), \end{array}$$

where Θ is a left-continuous unit step function, Γ_*in*_ and Γ_*out*_ are the respective sets of neighbors of the given node *i*, and $\frac{1}{n-1}$ is a simple normalization term (*n* − 1 is the number of possible incoming or outgoing edges a node can have in a network of *n* nodes). These values are plotted as coordinates in a Cartesian coordinate system, where the horizontal axis represents the total incoming CD of the area, and the vertical axis represents the total outgoing CD of the area (see **Figure 5B** for an example). The two axes divide the coordinate system into four quadrants and each area is represented in each quadrant according to Equations (6–9) as follows:

1. In the top left quadrant, the outgoing positive CD sums (Equation 8) are plotted as a function of the incoming negative CD sums (Equation 7) for each area.
2. In the top right quadrant, the outgoing positive CD sums (Equation 8) are plotted as a function of the incoming positive CD sums (Equation 6) for each area.
3. In the bottom left quadrant, the outgoing negative CD sums (Equation 9) are plotted as a function of the incoming negative CD sums (Equation 7) for each area.
4. In the bottom right quadrant, the outgoing negative CD sums (Equation 9) are plotted as a function of the incoming positive CD sums (Equation 6) for each area.

The quadrants represent different functional properties in terms of integration in the cortex: the combination of divergent input (negative incoming CD sum) and convergent output (positive outgoing CD sum) is, considering the information flow, equivalent to allocating information in the network. This is represented in the top left quadrant. In the opposite quadrant, the combination of convergent input and divergent output corresponds to source characteristics of the nodes (bottom right quadrant).

To further characterize each area with a single value, the *CD-flow* was defined as the difference in the means of incoming and outgoing edge CD-s (Bányai et al., 2011b).

(10) $$\begin{array}{l} \Phi \left(i\right)=\frac{1}{d_{out}\left(i\right)}\underset{j\in \Gamma_{out}\left(i\right)}{\sum }\text{CD}\left(i,j\right)-\frac{1}{d_{in}\left(i\right)}\underset{j\in \Gamma_{in}\left(i\right)}{\sum }\text{CD}\left(j,i\right), \end{array}$$

where Γ_*in*_ and Γ_*out*_ are the respective sets of neighbors of the given node, while *d*_*in*_ and *d*_*out*_ denote its in- and out-degrees. As the CD-flow is derived from the CD, it serves as a topological hierarchical index: nodes lower in the hierarchy have mainly convergent input and divergent output, while hierarchically higher nodes have divergent input and convergent output (Bányai et al., 2011b).

### 2.5. Randomization

To understand the organization of the macaque visuo-tactile cortex, its properties were compared to random graphs generated in two different ways. Erdõs-Rényi random graphs were constructed with the same number of nodes and edges as in the macaque visuo-tactile cortex. The other type of control network was generated by rewiring the visuo-tactile network without changing the degree distribution of the original cortical graph. Each calculation was performed on 30 random graph instances and the averaged graph properties are compared to the ones obtained from the visuo-tactile cortical network. It should be noted that the resulting randomized networks included a smaller ratio of reciprocal connections, than the visuo-tactile network. Basic properties of the visuo-tactile network and the randomized counterparts are summarized in Table 1.

**Table 1:** Graph metrics of the cortical visuo-tactile network and its randomized counterparts, the rewired and the Erdős-Rényi (ER) random graphs.

	**ER**	**Visuo-tactile**	**Rewired**
Density	0.33	0.33	0.33
Reciprocity	0.33 ± 0.02	0.77	0.44 ± 0.02
Diameter	3	3	3.27 ± 0.45
ASP	1.671 ± 0.002	1.78 ± 0.67	1.71 ± 0.01
EB max.	11.26 ± 1.68	43.1	44.65 ± 4.98
EB mean	5.02 ± 0.07	5.33 ± 4.4	5.14 ± 0.03
CI	0.55 ± 0.01	0.61	0.62 ± 0.01
λ_2_	8.27 ± 1.28	2.84	4.1 ± 0.05
λ_*max*_	21.64 ± 1.26	30.08	29.77 ± 0.1
λ_2_/λ_*max*_	0.38 ± 0.07	0.09	0.14 ± 0.002

*Mean ± sd are shown. In the randomized networks, standard deviation (sd) was computed from 30 graph instances. ASP, average shortest path; EB, edge betweenness; CI, global transitivity; λ_2_, algebraic connectivity; λ_max_, the largest eigenvalue; λ_2_/λ_max_, eigenratio*.

### 2.6. Edge Removal

Graph edges were attacked with two strategies: targeted and random removals. In case of targeted edge removal, links were successively eliminated according to the following criteria: descending order of edge betweenness, descending and ascending order of CD. The CD-based elimination was made by three different means: (i) minimum CD (minCD, divergent edges), (ii) maximum CD (maxCD, convergent edges), and (iii) absolute CD (absCD, representing the convergence/divergence potential of an edge). In targeted removal, all the relevant edge measures were recalculated after each removal and edges were deleted according to the recalculated values.

### 2.7. Weighted Shortest Paths

The standard Dijkstra's algorithm for finding weighted shortest paths works by minimizing the cost of traveling a sequence of edges between the starting and ending nodes. The cost of traversing an edge is generally taken to be the reciprocal of its weight. Notably, this algorithm does not take into account the number of edges (i.e., jumps) in a path, thus it returns shortest paths in which the cost is minimized, but the number of jumps might be quite high. Depending on the network, this may lead to shortest path structures that are not accurate representations of the most efficient communication channels (Opsahl et al., 2010; Avena-Koenigsberger et al., 2018). One option for resolving this problem is the inclusion of an extra parameter in computing the costs: exponent α, quantifying a trade-off between the importance of the weights and the number of jumps (Opsahl et al., 2010). The shortest path between nodes *i* and *j* is then:

(11) $$\begin{array}{l} d\left(i,j\right)=min\left[\frac{1}{w_{ik}^{\alpha }}+\ldots +\frac{1}{w_{lj}^{\alpha }}\right], \end{array}$$

where *w*_*xy*_ is the weight of the edge (*x, y*), *k*…*l* are indices of the areas in the path and the exponent α is the tuning parameter. It can take any real positive value, but if α = 0, then the weights lose influence and Dijkstra's algorithm returns the binary shortest paths, while α = 1 returns the standard procedure where the number of jumps is neglected; between 0 and 1 the trade-off is manifested.

Another problem with the standard approach for finding weighted shortest paths is purely numerical. In fact, binary graphs can be understood as a subtype of weighted ones, where the path costs are “binned,” that is, they are integers, instead of real numbers. Since integers provide much smaller variability in a given interval, binary path lengths will fall into only a few “categories,” i.e., many of them will have the same length. This leads to the fact that in a binary graph usually there are several shortest paths connecting any given node-pair. In weighted graphs, on the other hand since the weights (and therefore the costs) are real numbers, it is extremely rare that two path lengths coincide, resulting in a shortest path structure that relies on a few very popular edges. A side effect is that most of the edges are excluded from the communication in the graph. There are a few options for resolving this problem, one being to take multiple, i.e., k-shortest paths between every node pair instead of only one (Avena-Koenigsberger et al., 2017).

### 2.8. SLN-Based Anatomical Hierarchy and Hierarchical Distance

Within an area the ratio of labeled supragranular layer projection neurons (SLN) quantifies the hierarchical properties of an edge in the large-scale anatomical network (Markov et al., 2014). Specifically, an edge with a zero SLN value is assumed to represent only feedback axonal projections, while an edge with a maximal SLN of 1 is assumed to represent solely feedforward projections. Any value between signifies a ratio of FF and FB projections comprising that edge. Note that SLN is not complementary for reciprocal edges, there can be FF- and FB-type communication in both directions (e.g., between areas at nearby hierarchical levels). Following Markov et al. (2014), the hierarchical level of an area, based on the SLN values of its edges, was computed by fitting a generalized linear model with a beta-binomial distribution and a logit link function [note that Markov et al. (2014) actually used a probit link, but as they point out the two link functions give very similar results]. Refer to section 2 of the Supplementary Methods and to Markov et al. (2014) for further details on the implementation.

In addition, the (anatomical) hierarchical distance was defined as the pairwise difference in the SLN-based hierarchical levels of areas. The sum of hierarchical distances (SHD) for a given subnetwork equals to the summation of all pairwise hierarchical differences between areas that make up the subnetwork.

### 2.9. Multi-Scale Dynamical Model

In order to investigate the relation between network structure and dynamical interactions, a Wilson–Cowan-type multi-scale dynamical model was implemented following closely (Mejias et al., 2016). The model describes four embedded levels of neuronal structure: (i) a mutually connected pair of local excitatory and inhibitory populations forming the basic circuitry of a given cortical layer, (ii) a macroscopic cortical area is considered as a bilaminar structure modeled by the interconnected basic laminar circuitry representing the supragranular and infragranular layers, (iii) inter-areal interactions were modeled by connecting the bilaminar circuitries of two areas in a way that represents the hierarchical laminar distribution of the feedforward and feedback anatomical connectivity, and (iv) the large-scale cortical network was modeled as the interconnected 29 areas, mainly in the visual pathways.

The coupling of the supragranular and infragranular circuits was tuned to oscillate in the gamma and alpha-beta range, respectively. In laminar interactions, supragranular to infragranular connections originated in, and targeted excitatory populations, whereas infragranular to supragranular excitatory connections targeted inhibitory populations. Inter-areal connections were excitatory to model associational connections of the pyramidal cells. Feedforward (FF) interactions were formed between the excitatory populations of the supragranular layers. In contrast, feedback (FB) excitatory connections targeted both excitatory and inhibitory populations of the supra- and infragranular layers. The ratio of FF and FB communication upon a directed edge was determined by the ratio of supragranular labeled projection neurons, SLN (SLN = 1 for solely FF, SLN = 0 for solely FB, and a mixture of the two in-between). The overall strength of inter-areal connections was determined by the fraction of labeled projection neurons, FLN, and delay was added to the coupling terms according to the physical distances of the projections. This architecture reproduced the experimentally observed bilaminar large-scale hierarchical dynamics of cortical interactions with feedforward evoked gamma-range oscillation in the supragranular layer and feedback induced alpha-beta-range oscillation in the infragranular layer (Bastos et al., 2015). A detailed description of the model including a table with the parameters used (Supplementary Table 1) is provided in section 1 of the Supplementary Methods.

The simulated time series were used to compute the conditional spectral Granger causality and the functional connectivity in form of a frequency-dependent, directed asymmetry index (DAI, detailed below) between nodes, as described in Mejias et al. (2016), main text and Supplementary Methods.

### 2.10. Conditional Spectral Granger Causality Analysis

Granger causality (GC) quantifies the directed influence between two processes, based on the ability to predict future values. In particular, if the accuracy of predicting future values of process X is improved by including information about process Y, with respect to the prediction considering solely values of X itself, then one may say that Y “Granger causes” X. The spectral version of the GC analysis works in the frequency domain instead of the time domain. Conditional spectral GC is needed when one wishes to compare more than two frequency spectra because a pairwise comparison would include indirect effects from other sources, making the comparison unreliable (Wen et al., 2013). Refer to Wen et al. (2013) (Equations 2.22–2.36) for the exact formulation used in the present work. The model we used was focused on GC in the gamma and alpha-beta frequency ranges.

### 2.11. Directed Influence Asymmetry Index (DAI)

Based on findings that show the layer-specificity of synchronization frequency of cortical interactions, Bastos et al. (2015) defined an index of functional hierarchy, called directed influence asymmetry index (DAI). The DAI is the normalized difference of the conditional Granger causality (GC) spectra taken in the two directions between a pair of time series recorded from a pair of cortical areas:

(12) $$\begin{array}{l} {\text{DAI}}_{s\to t}\left(f\right)=\frac{{\text{GC}}_{s\to t}\left(f\right)-{\text{GC}}_{t\to s}\left(f\right)}{{\text{GC}}_{s\to t}\left(f\right)+{\text{GC}}_{t\to s}\left(f\right)}, \end{array}$$

where the indices *s* and *t* signify the source and target area, respectively. Note that DAI_*s*→*t*_(*f*) = −DAI_*t*→*s*_(*f*). The DAI is a spectrum itself, and Bastos et al. (2015) found that DAI values exhibit positive correlation with the anatomical hierarchical index SLN in the gamma- and theta-frequency bands, and negative correlation in the alpha-beta-band, in the 8 × 8 subnetwork they measured. A point estimate of the DAI spectrum is the multifrequency-band DAI (mDAI), which is calculated by averaging the DAI of the theta, beta and gamma ranges (after inverting the beta values because of their negative correlation with the SLN). Note that following (Mejias et al., 2016) the theta band was not included in the analyses due to its minuscule influence, therefore the mDAI is computed as:

(13) $$\begin{array}{l} {\text{mDAI}}_{i\to j}=\frac{{\text{DAI}}_{i\to j}\left(\gamma \right)-{\text{DAI}}_{j\to i}\left(\alpha \right)}{2}, \end{array}$$

where the DAI for a given frequency range ω is:

(14) $$\begin{array}{l} \text{DAI}\left(\omega \right)=\int_{\omega_{min}}^{\omega_{max}}\text{DAI}\left(f\right)df, \end{array}$$

where the alpha/low-beta range was taken to be 6–18 Hz, while the gamma range was 30–70 Hz. Bastos et al. (2015) found that the mDAI correlates well with SLN in the studied 8 × 8 subnetwork, and Mejias et al. (2016) showed that all correlations can be reproduced by their multi-scale dynamical model applied in the present study.

### 2.12. Implementation

For computations and illustrations concerning efficiency and synchronizability, the Octave and the R environment was used with the addition of the free *igraph* package^2^ for graph generation, randomization, and shortest path calculation (Csárdi and Nepusz, 2006). For the relaxed shortest paths, the weighted convergence degree and dynamical simulations a Python environment was used with the freely accessible packages Numpy, Scipy, Pandas, Matplotlib, and Networkx. The dynamical model of Mejias et al. (2016) was implemented in Python with the help of their Matlab code that can be reached on ModelDB^3^. The conditional spectral GC analysis had to be implemented in Python, which was done based on Wen et al. (2013) and relying on the framework of the *spectral connectivity* package^4^.

## 3. Results

### 3.1. Network Efficiency and Synchronizability Depend Differently on Edge Betweenness and Convergence Degree

#### 3.1.1. Effects of Edge Removal on Structural Integrity and Global Efficiency

**Figure 1:**
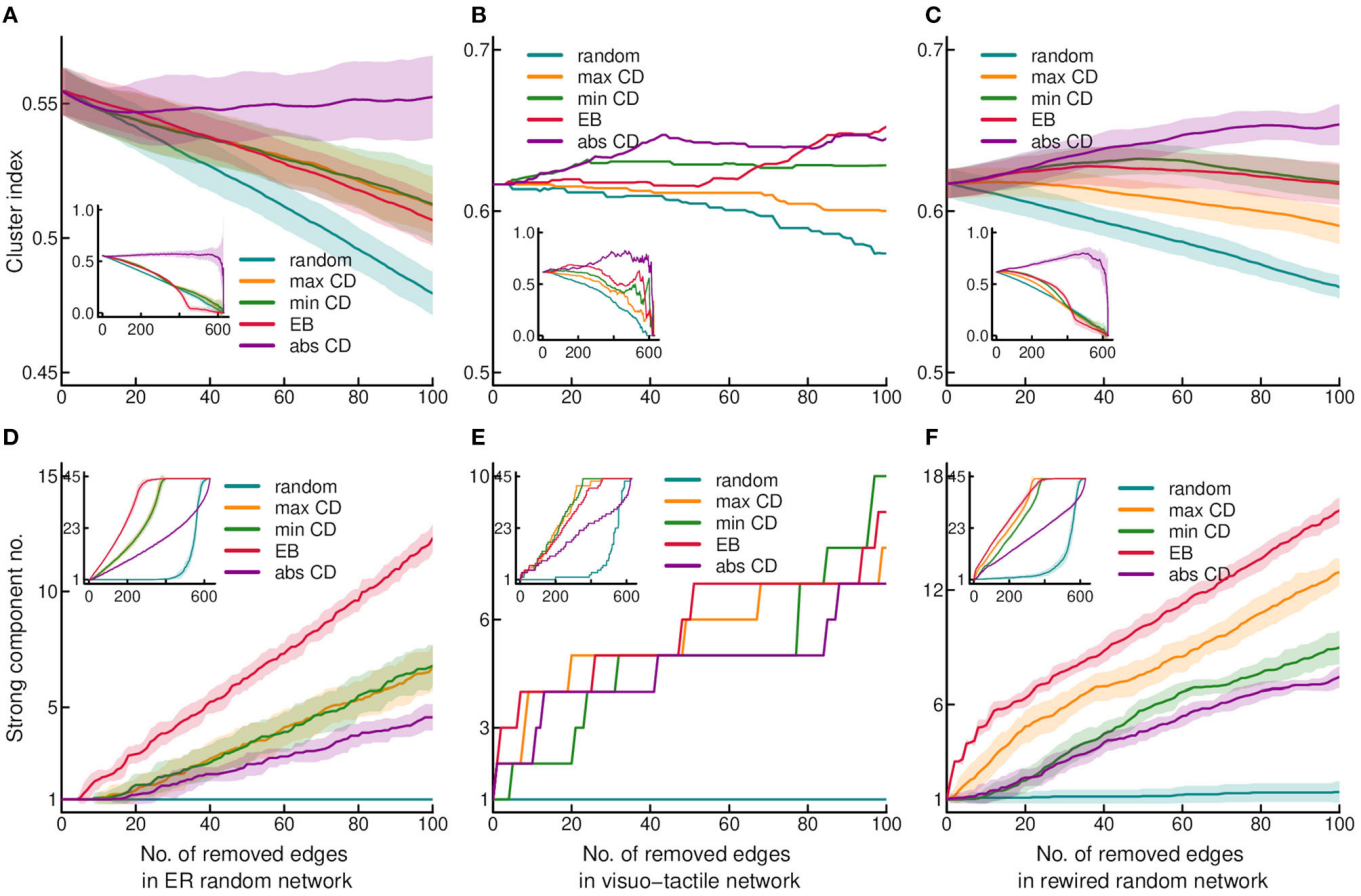
Effect of edge removal on the cluster index and strong connectedness. Cluster index and the number of strongly connected components are shown as the function of stepwise edge removals in the upper **(A–C)** and lower **(D–F)** rows, respectively. Middle column shows results obtained in the binary visuo-tactile network **(B,E)**. Left and right columns show the results obtained in the Erdős-Rényi (ER) random graphs **(A,D)** and rewired randomized control networks **(C,F)**, respectively. The graphs show the result of the first 100 edge removals, while the insets contain the process for the entire graph. Edge properties were recomputed after each step of removal. In randomized networks, the average of 30 graph instances is shown with shading indicating the standard deviation. Targeted edge removal was executed according to maximum CD (maxCD), minimum CD (minCD), absolute CD (absCD), and edge betweenness (EB) values. The results of random edge eliminations are also shown for reference.

**Figure 2:**
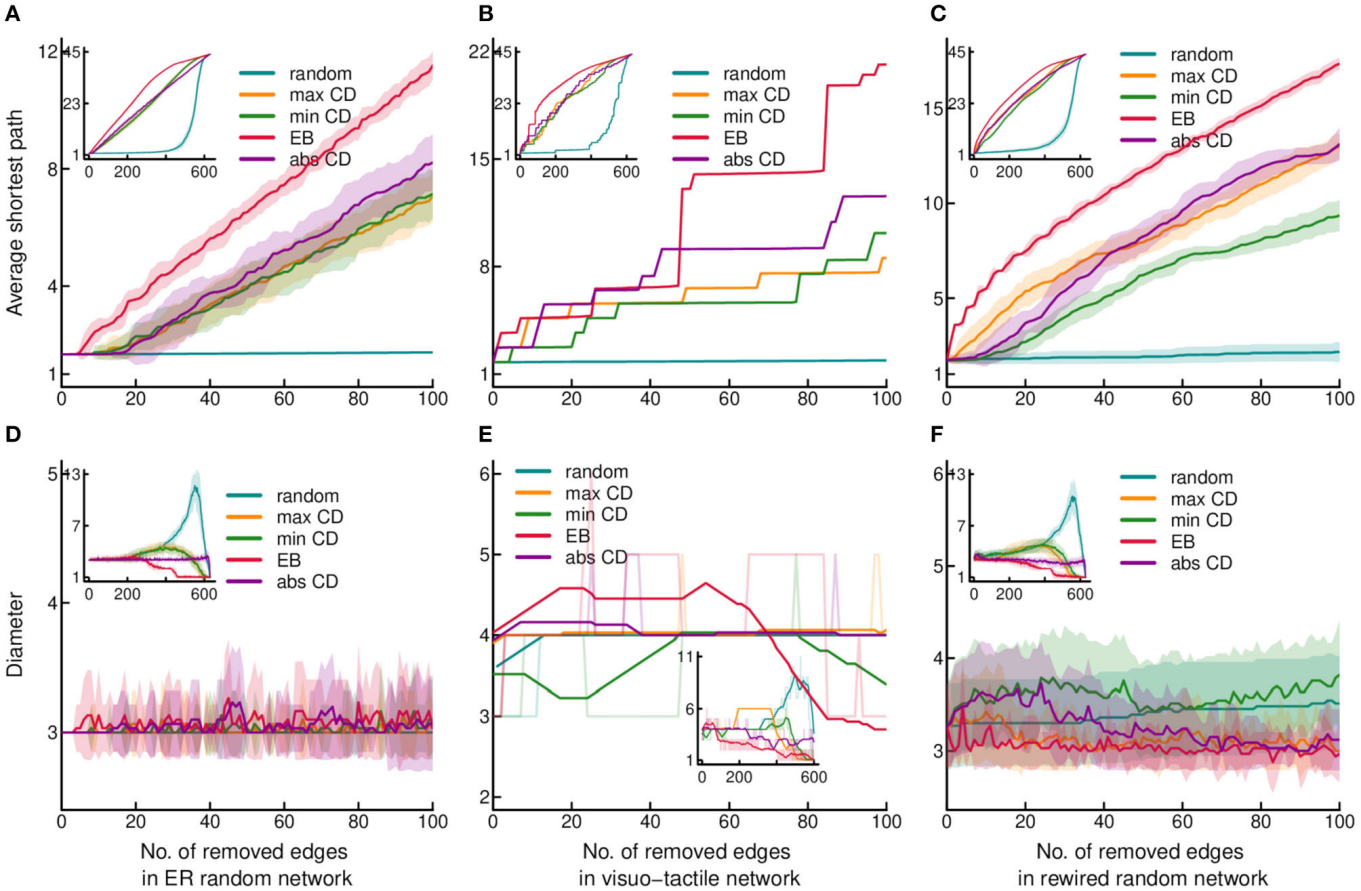
Effect of edge removal on global network efficiency. **(A–C)** present changes of the average shortest path, while **(D–F)** show changes of the diameter, as the function of stepwise edge removals. Changes in diameter were smoothed by moving average with a 31 step window size. However, the non-smoothed original data is also indicated by the lighter coloring of the lines. All the conventions and the layout of the plots are the same as in Figure 1.

**Figure 3:**
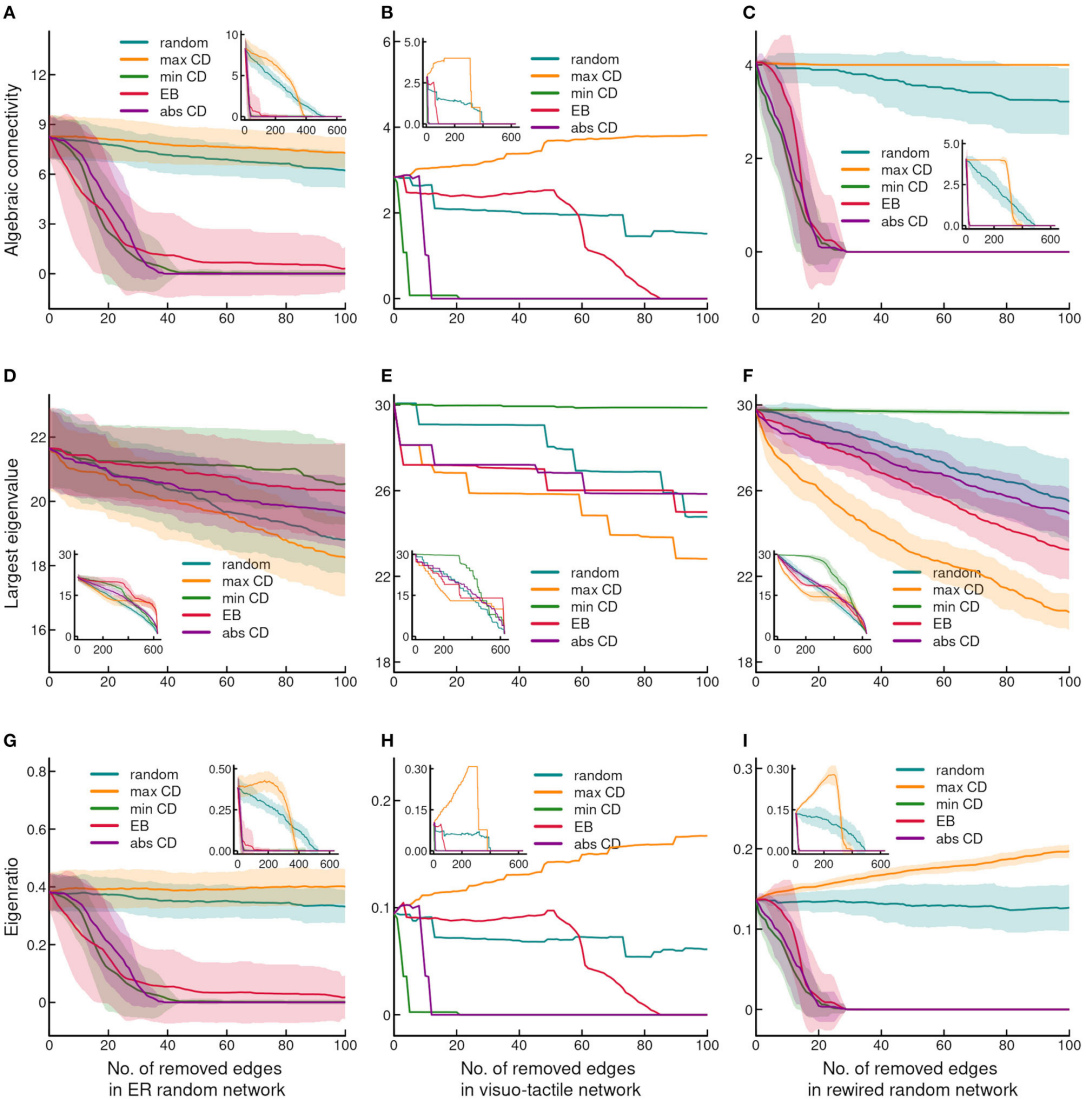
Effect of edge removal on network integrity. **(A–C)** shows changes of the algebraic connectivity, **(D–F)** illustrate changes of the largest eigenvalue and **(G–I)** demonstrate changes of the eigenratio, as the function of stepwise edge removals. Note the almost identical pattern of changes of the algebraic connectivity and the eigenratio. The layout of the plots and the conventions are the same as in Figure 1.

As a general observation regarding the results of the role of edge properties on network resilience, there appeared to be a broad overall similarity of the pattern of changes between the cortical and control networks upon edge removal (Figures 1–**3**). This finding indicates the generality of the studied network properties. However, there were notable differences between the different networks, which indicate the dependence of edge property on the specificity of network architecture. Our analysis was focused on the differences in vulnerability observed for the different networks.

First, we were interested in how quickly the cortical network disintegrates upon edge removal by different strategies. To this end, the effect of attack was tested on global transitivity (based on the cluster index, CI) (Figures 1A–C) and strong connectedness (Figures 1D–F). In the cortical network, CI was found very robust against edge removal with the highest sensitivity to random attack (Figure 1B). Compared to the cortical network, control networks were more vulnerable with the highest sensitivity to the random attack, similarly as found for the cortical network at least in the initial phase of edge removal (Figures 1A,C). The CI can be especially sensitive to the level of reciprocity, as it is computed by ignoring directedness (i.e., disconnecting the triangles requires removing both of the reciprocal edges). The CI was the highest in the cerebral cortex (Table 1). Since clustering is proportional to the number of triangles in the network, this finding also pints on the importance of connected community structures of the cortical network, which cannot be explained by its degree distribution. Also, considering the balanced magnitude of convergence and divergence via the reciprocal connections (Négyessy et al., 2008), the lack of effect of absolute convergence degree (absCD) on CI suggests that removing one of a reciprocal link results in the decrease of absCD of the remaining link, i.e., the reorganization of the CD properties among the remaining edges.

In contrast to CI, strong connectedness was very sensitive to targeted edge removal in the cortical network, which increased the number of disconnected components (i.e., in terms of strong connectedness) right after deleting the first few edges (Figure 1E). Strong connectedness was equally sensitive to targeted removal based on edge betweenness (EB) and CD with a smaller effect of absCD, but very robust against random attack. In contrast to the cortex, randomized networks showed high vulnerability of strong connectedness exclusively to EB-based elimination (Figures 1D,F). Although, comparing the control networks, it should be noted that the rewired network behaved more like the cortical than the Erdõs-Rényi (ER) random network upon edge removal, which indicates the higher vulnerability of networks with non-homogeneous degree distribution (Figures 1D–F). In the case of strong connectedness, reciprocity can also play an important role as a large number of random attacks were probably needed to eliminate both links of a reciprocal pair. However, in strong connectedness the large effect of EB indicates the additional role of high transmission links, which usually connect network clusters. Based on the sensitivity of strong connectedness to attack against edges, here and in the following we graphically represent the results of removal of the first 100 edges, while the results of removing all the edges will be shown in the insets of the figures.

The average shortest path (ASP) as a measure of global efficiency exhibited similar sensitivity to edge removal as found for strong connectedness with the highest vulnerability shown by the cortical network (Figures 2A–C). Also, regarding the control networks, ASP of the rewired network was affected similarly by edge removal to that of the cortical network while the ER network exhibited higher robustness (Figures 2A–C). The ASP was affected strongly by targeted attack and was resilient to random edge removal (Figures 2A–C). Among the edge properties, ASP was the most sensitive to EB-based elimination. Contrary to what was observed on measures of network integrity (Figure 1), which was vulnerable to the removal of both of the convergent and divergent connections but was resilient to attacking edges based on absolute CD, ASP was vulnerable to targeting edges irrespective of the links' CD properties including absCD. This result shows that ASP was not sensitive to the convergence properties of the connections.

Diameter, the other index used as an indicator of global efficiency, exhibited similar changes following edge removal to that found in the case of strong connectedness and ASP (Figures 2D–F). Accordingly, diameter decreased the most after targeted attack by EB. In contrast, random attack resulted in a different pattern by increasing the diameter due most probably to the increase of the size of connected components which disconnects in the later phase of edge deletion. That network efficiency was mostly dependent on EB suggest the importance of clustering, which is the highest in the cortical network (Table 1), as inter-cluster links are usually high traffic “short cuts.”

#### 3.1.2. Effects of Edge Removal on Synchronizability

The spanning of eigenvalues was the largest in the cortical network exhibiting the smallest λ_2_ and the largest λ_*N*_ (Table 1). In contrast, the range of eigenvalues was the smallest in the ER random network. The λ_2_ and λ_*N*_ of the rewired network differed less from those of the cortical than from the ER network, which indicates the importance of degree distribution, and the diversity of connectedness in the spectral properties of the graph (Barahona and Pecora, 2002; Nishikawa et al., 2003; Chen et al., 2012). These findings suggest that compared to the ER network, which reaches a stable synchronous state easily, the architecture of the cortical network supports a relatively slowly emerging but richer pattern of synchronous oscillations (Almendral and Díaz-Guilera, 2007; Arenas et al., 2008; Chen et al., 2012). Also, the small λ_2_ suggests that cortical network is sensitive to bottleneck properties, i.e., structural and functional disintegration upon attack (Boccaletti et al., 2006), which is consistent with the vulnerability as shown for indices of network efficiency and strongly connected component (Figures 1D–F, 2).

The most remarkable difference of vulnerability between the cortical and control networks appeared in the case of synchronizability measured by λ_2_ (used in the plots as algebraic connectivity) (Figure 3). Synchronizability was affected the most by removing edges in the order of their minCD (Figures 3A–C). In the cortex, synchronizability dropped to near zero after removing just a few edges based on minCD, which indicates the appearance of a strong bottleneck effect in the network through divergent links. Although with some lag compared to that seen for minCD, the cortical network was also vulnerable when targeting edges according to absCD, which suggests the sensitivity of the magnitude and/or sign of CD to edge removal similar to that seen for the cluster index. Interestingly, in term of synchronizability the cortical network was more robust to EB-based deletion than both of the control networks. This finding suggests that bottlenecks are not necessarily the links with the highest traffic as determined by EB due to existing alternative routes. Instead, our findings show the importance of the impact of nodes on each other through the directed paths in the networks. Most notably, it seems that global divergence is an especially important network property by determining the strength of influence (i.e., “sourcness”) via the links. In other words, cutting away sources have a highly devastating effect on network interactions as shown by synchronizability.

In contrast to algebraic connectivity, the largest eigenvalue was relatively resilient to targeted and random edge eliminations (Figures 3D–F). However, it was notable that in the cerebral cortex targeting edges by their minCD and maxCD values had the opposite effect on the largest eigenvalue then seen in the case of algebraic connectivity. The largest eigenvalue was highly robust to edge removal by the minCD in the cortical network. In addition, although, the highest vulnerability was found in maxCD-based attacking, the effect of EB-based removal exhibited similar susceptibility of the largest eigenvalue (Figure 3E). Also, random and absCD-based attacking resulted in relatively small differences of the change of the largest eigenvalue from that observed following maxCD-based link elimination. In the ER graph, all edge targeting strategies exhibited a similar effect on the largest eigenvalue (Figures 3D–F). A further notable difference was the relatively high resilience of the largest eigenvalue to EB-based link targeting in the ER than in the other two networks. Accordingly, in the cortical and rewired networks the similarity of changes of the largest eigenvalue compared to that seen in the ER network indicated the dependence of this topological measure on the heterogeneity of degree distribution (Figures 3D–F). Finally, the eigenratio was determined by the higher vulnerability of algebraic connectivity than the largest eigenvalues, and exhibited almost exactly the same pattern of changes as shown for algebraic connectivity (Figures 3G–I).

It should be noted that in contrast to measures related to global efficiency, graph spectral values, especially synchronizability and eigenratio were affected differently by edge removal in the cortical network and its randomized counterparts including both the rewired and ER networks. This observation is in accordance with the nature of graph metrics as eigenvalues uncover unique, distinguishing topological features of the networks unlike the other more simple indices used here.

### 3.2. Role of CD in Hierarchical Network Dynamics

#### 3.2.1. A Relaxed Weighted Shortest Path Structure

To compute the weighted shortest paths of the graph the cost was defined as the product of the projection distance in millimeters (the “length” of the edge) and the reciprocal of the FLN (fraction of labeled projection neurons; the “strength” of the edge). Thus, for a given connection, its distance was directly, while its FLN value was inversely proportional to the cost of traversing it. Note that neither the SLN (ratio of the supragranular labeled projection neurons) nor any other explicit hierarchical information was incorporated in the cost.

**Figure 4:**
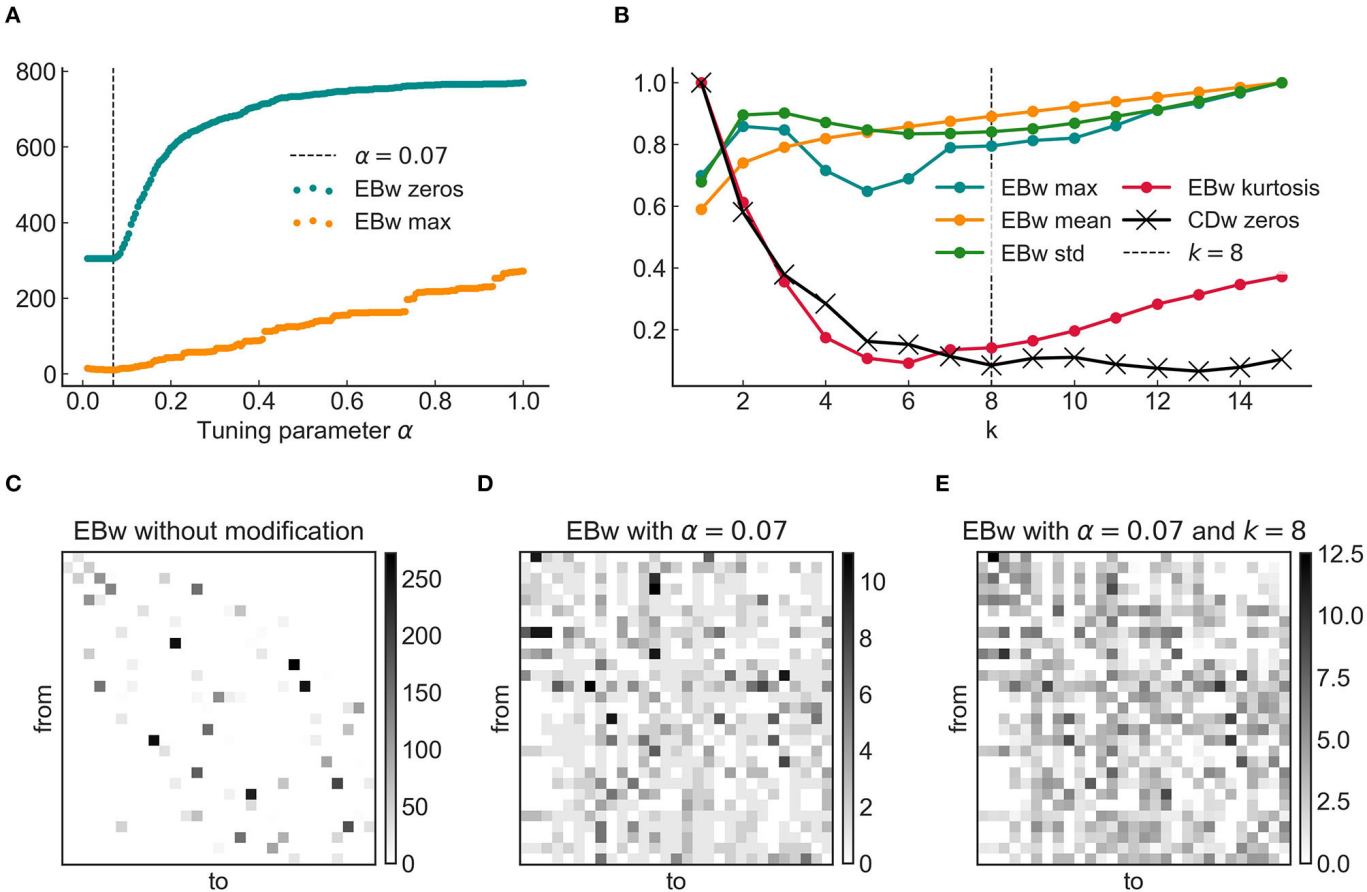
Optimization of the parameters α and *k*. **(A)** The number of unused edges (those with a zero weighted edge betweenness, or EBw value) and the exclusive popularity of a few edges (the maximum of all EBw values) were minimized while keeping the significance of the weights (α) at the highest value possible. **(B)** Normalized EBw statistics and number of edges with a zero weighted convergence degree (CDw) as a function of *k*, i.e., the number of shortest paths considered for each pair of nodes. The maximum, mean and standard deviation of the EBw did not change considerably. The kurtosis fell to a minimum around *k* = 6 and started to increase again, while the number of zero CDw values reached a minimum around *k* = 8 and did not change significantly afterwards. Jointly minimizing the kurtosis of the EBw and the number of zeros in the CDw resulted in an optimal parameter value *k* = 8. **(C–E)** Three adjacency matrices showing the EBw values as weights. **(C)** Without the extra parameters, most edges were unused and the workload was unrealistically huge on a few super-popular edges. **(D)** The shortest path structure resulting from the optimal α had a much more reasonable distribution. The number of zeros was the lowest possible (white cells in the matrix are absent edges), although most edges supported only a single shortest path (i.e., the edge itself connecting the two nodes at its ends). **(E)** Applying the optimal *k* distributed the communication load more evenly by placing more weight on barely frequented edges.

Applying the standard Dijkstra's algorithm to these weights resulted in shortest paths that take long detours (sometimes up to 20 jumps) in pursuit of minimum cost instead of taking the direct, although more expensive route. This is clearly unrealistic since the signal arriving into an area has to pass through a myriad neuronal somas, axons, and synapses, all of which impose a delay on its flow that cannot be neglected respective to the relatively quick propagation of action potentials along interareal axonal bundles. The algorithm also returned a very sparse shortest path structure, with only a few extremely popular edges being responsible for the entirety of the signal flow upon the graph (Figure 4C).

To resolve these problems, a major development of our work was the joint employment and optimization of two extra parameters in finding the shortest paths in a cortical graph. The α tuning parameter was added to the cost as an exponent, and its value determined a trade-off between the weighted cost (i.e., the strength of edges) and binary cost (i.e., the number of jumps). This way, the shortest weighted path between areas *i* and *j* can be found as:

(15) $$\begin{array}{l} d\left(i,j\right)=min\left[{\left(\frac{{\text{FLN}}_{ik}}{dist_{ik}}\right)}^{\alpha }+\ldots +{\left(\frac{{\text{FLN}}_{lj}}{dist_{lj}}\right)}^{\alpha }\right], \end{array}$$

where *dist* is the distance in millimeters and *k* … *l* are indices of nodes making up the path. To find the optimal value for α both the number of zero EBw values (edges excluded from the signal flow) and the highest EBw (the burden on the most popular edge) were minimized while keeping α the highest possible (to maintain the influence of the empirical weights). Figure 4A shows this optimization process, which resulted in the optimal α = 0.07, assigning much more cost to jumps than to weights. Any value greater than that brings us back into the realm of long detours, while lower values discard the information of weights.

The resulting shortest path structure was much closer to what one would expect for a real-world network, although the number of shortest paths was still less than a third of those found in the binary case (812 against 2,903), which itself has a sparse structure. This can be observed in Figure 4D, as a prevalence of edges that support only a few shortest paths, and only a few that are 4-5 times more popular. In fact, at this point moderately meaningful results can already be computed for the weighted convergence degree (CDw), although due to the small number of shortest paths, and the large number of edges with only one shortest path (i.e., the edge itself), the CD will give a lot of zero values (both the in- and out-set having only one element). This is not the case for the CD computed on the binary graph (CDb) which shows very few zero values, nor it is in line with previous findings that show a very hierarchical order based on the CD, with a minimal number of balanced edges.

These concerns were addressed by considering not a single, but multiple, i.e., *k* shortest paths for each node-pair. This method also increases robustness in the face of damage or malfunction in the network structure, which is an observed feature of many real-life networks, including the brain (Avena-Koenigsberger et al., 2017, 2018). Investigating the statistics of the EBw distribution in response to changes in *k*, the kurtosis of the distribution emerged as the only statistic that changed appreciably. This should not be surprising since more alternative shortest paths reduce the load on the most popular edges by dividing the communication among other edges, thus decreasing the “tailedness” of the distribution (Figure 4B).

In response to these findings, optimization was done by joint minimization of the kurtosis and the number of zero CDw edges, resulting in the optimal parameter value *k* = 8 (Figure 4B). With α and *k* chosen this way, this *relaxed* weighted shortest path structure became similar to the binary case (i.e., it has several alternative but similarly short paths between any two nodes), while also taking into account the empirical characteristics of cortical projections (in the form of empirical weights; Figure 4E).

#### 3.2.2. Analysis of the Weighted CD

**Figure 5:**
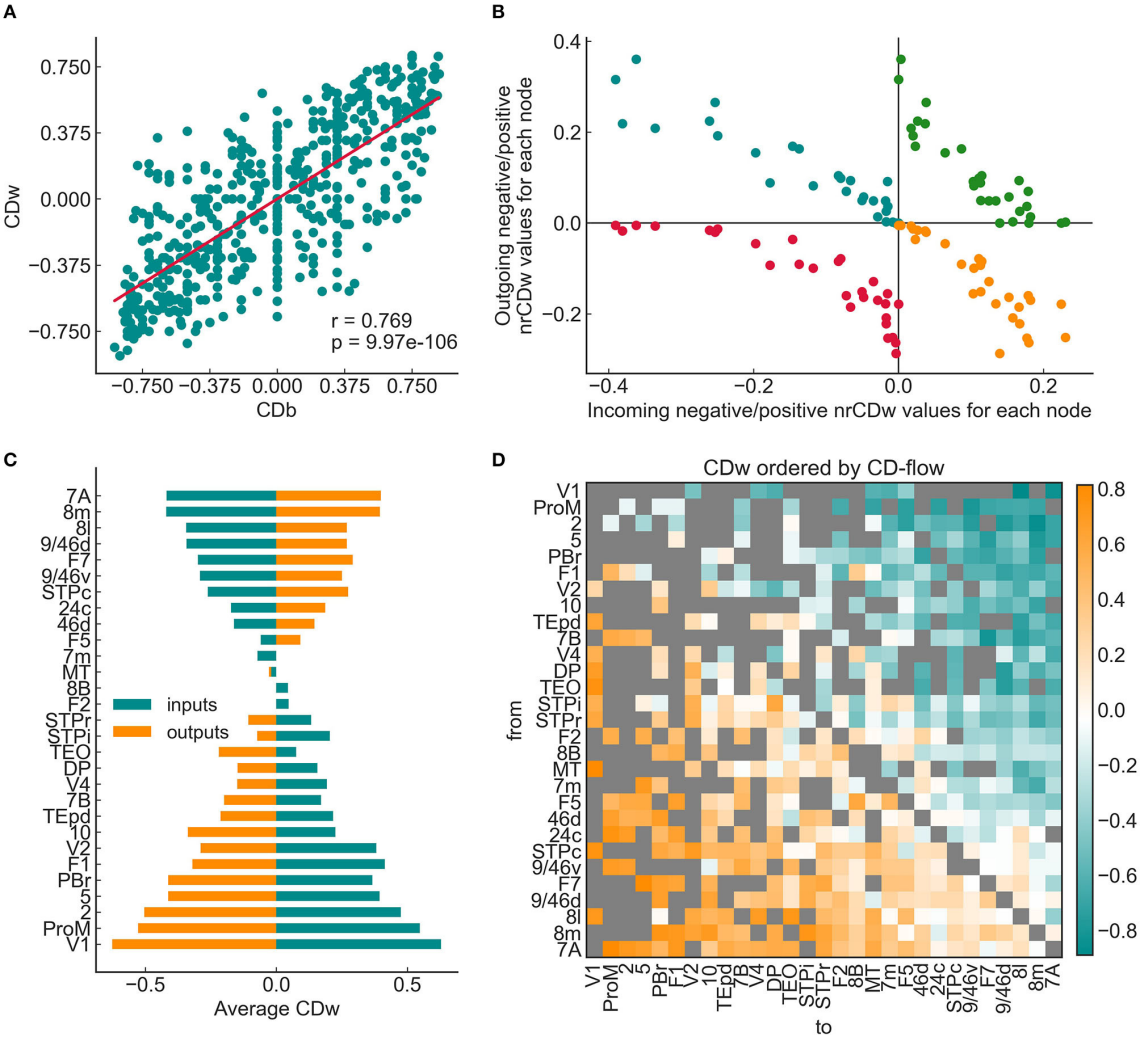
Weighted convergence degree of the 29 × 29 graph. **(A)** Linear correlation between the binary and weighted convergence degree values (CDb and CDw, respectively). The binary graph had a relatively sparse shortest path structure, leading to a higher probability for zero CDb values. The weighted CD appeared more “fine-grained,” i.e., most edges have a unique CDw value. **(B)** Weighted node-reduced CD (nrCD) plot following the convention of Négyessy et al. (2008) and Bányai et al. (2011b). Note that every node is present in all four quadrants. The (–,+) and (+,–) quadrants show a clear negative correlation, while the other two quadrants show hyperbolic-like distributions, signifying a highly hierarchical composition. **(C)** Average incoming and outgoing CDw values for the entire graph. The areas are ordered by the CD-flow from the bottom-up. The same hourglass shapes can be seen as was reported by Négyessy et al. (2008), with mostly source areas residing in the bottom half and allocating areas in the top half. **(D)** CDw values for the weighted 29 × 29 graph, ordered by the CD-flow, revealing several interesting details about the hierarchical organization of the signal flow structure. Most striking is the almost perfect gradient, perpendicular to the diagonal, moving from extremely convergent (orange) to extremely divergent (blue) edges, with the zero CD edges mostly aligning in the middle, along the diagonal. Another clear feature is a densely connected, rich club-like cluster of higher-order areas in the bottom right quadrant, in stark contrast with lower areas that exhibit a much sparser connection pattern. Note that neither the edge betweenness nor the experimentally measured SLN shows such structure.

The relaxed weighted shortest path approach described in the previous section allowed the exploration of the degree of convergence/divergence in the weighted cortical network for the first time. First we computed the binary (CDb) and the weighted convergence degree (CDw) of the graph. The joint distribution of CDw and CDb showed a highly significant positive correlation according to the expectations since the binary network serves as a backbone for the weights defined in the previous subsection. Importantly, CDw exhibited a much more refined distribution than the CDb, due to the larger number of shortest paths detected by the relaxed shortest path approach, thus providing a more realistic representation of the convergence degree in the weighted than in the binary cortical network (Figure 5A).

For the complete understanding of CD characteristics of the weighted network, the node-centric convergence degree measures (nrCD and CD-flow) were also computed. Figure 5B shows the nrCDw representation of the graph, which presents the characteristic shape shown by Négyessy et al. (2008) and Bányai et al. (2011b). Every node is present in all four quadrants, quantifying different aspects of the CD-based signal-coordinating capacity of the area the given node represents in the network. In the (–,+) and (+,–) quadrants nodes align clearly in a negative correlation between allocating and source attributes, respectively. Area V1, which is on the bottom end of the CD-flow hierarchy (totally source), is close to the origin in the (–,+) and furthest from the origin in the (+,–) quadrant. The relay attributes of the nodes represented in the (–,–) and (+,+) quadrants show hyperbolic-like shapes, signifying a minimized relay character and therefore a markedly hierarchical organization of the signal flow structure. Notably, the weighted version of the nrCD plot is a more specific representation with fewer outliers than its binary version (not shown).

Figure 5C shows the average incoming and outgoing CD values of nodes, which were ordered by the CD-flow. The observable hourglass shape also corresponds well to that reported previously (Négyessy et al., 2008). According to the CD-flow ranking, the hourglass shape signifies a gradient from source to allocating nodes, in the bottom-up direction similarly as found in the binary network (Négyessy et al., 2008). These observations (Figures 5B,C) supported the usefulness of CDw as an index of topological hierarchy in the weighted network of the cerebral cortex.

Visualized by an adjacency matrix with areas ordered by the CD-flow (Figure 5D) the asymmetric distribution of CD-s is salient, with mainly convergent edges in the lower triangle, divergent edges in the upper triangle and a gradual change through more neutral CD-s between these two extremes. Remarkably, a clear tendency can be discerned with higher-order areas forming significantly more connections than lower-order areas. This clusterization resembles a rich club that was not reported in the case of SLN and adds further support for the reliability of using CDw in exploring the unique topological properties of the cerebral cortex (van den Heuvel and Sporns, 2011).

#### 3.2.3. Correlations of Topology, Anatomy, and Dynamics in the 8 × 8 Susbgraph

**Figure 6:**
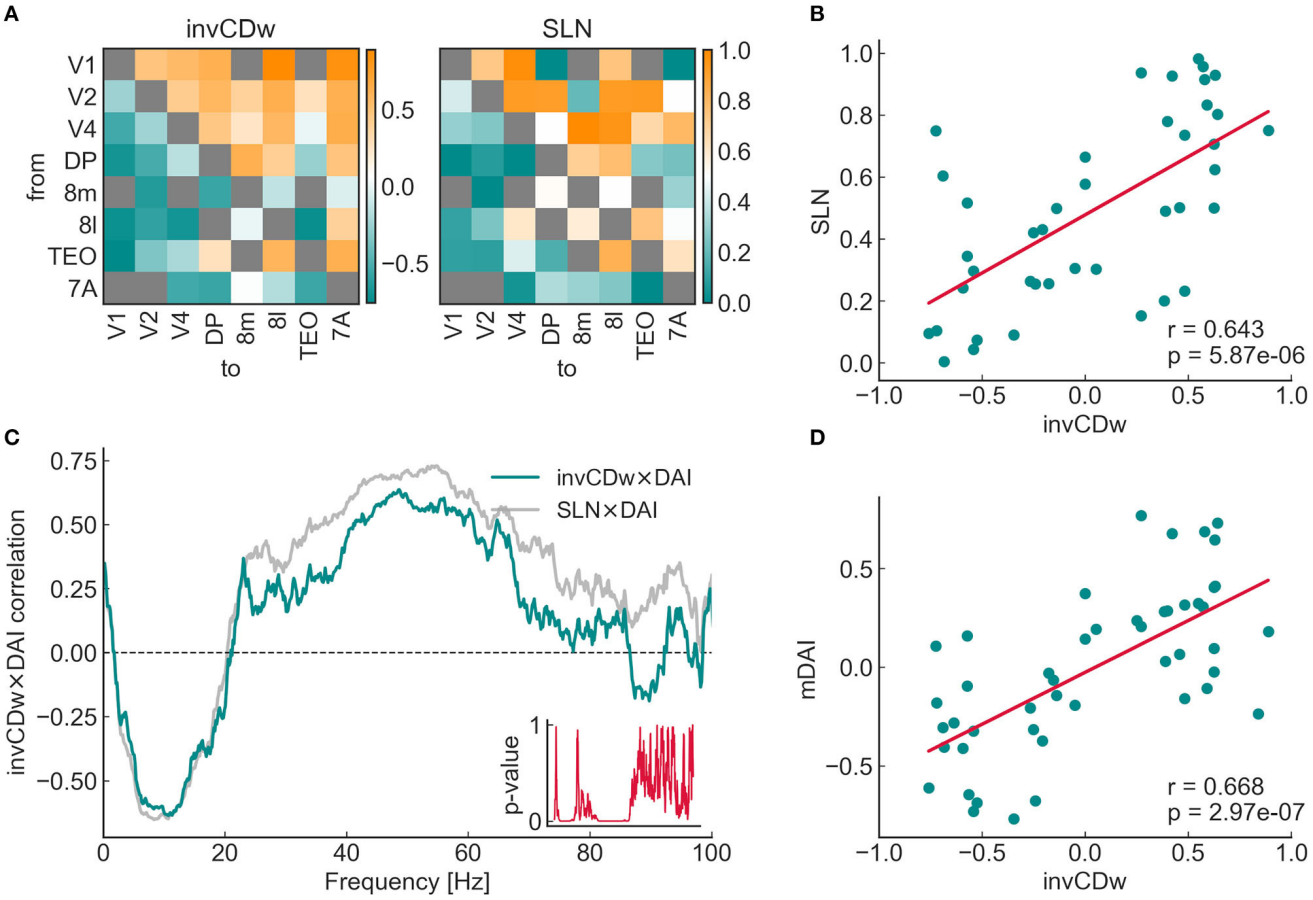
Relationship of the additive inverse of the weighted convergence degree (invCDw) with the SLN (fraction of supragranular labeled neurons) and the DAI (directed influence asymmetry index, see the main text for complete descriptions) in the 8 × 8 subgraph. **(A)** The invCDw and the SLN matrices of the 8 × 8 subgraph, the latter based on the data from Markov et al. (2014), as in Mejias et al. (2016). Both distributions are highly asymmetric. Both are ordered according to the anatomical (SLN-based) hierarchy, therefore it shows that for this subgraph, convergent edges highly coincide with feedforward connections (upper triangle from the diagonal), while divergent edges coincide with feedback connections (lower triangle). It is also clear just by looking at the color distributions, that area TEO would be much lower, while frontal eye field areas 8m and 8l would be higher according to the CD-based ranking. Gray cells are NaNs, where there is no edge in the graph. **(B)** There is a significant positive correlation between the SLN and the invCDw (*p* < 10^−5^). Note that invCDw was derived solely from the topology of the weighted network; neither SLN, nor DAI were used in its calculation. Note also that except omitting extreme SLN values (0 and 1) similarly to Mejias et al. (2016), no outlier exclusion was done in any of the plots in this work. **(C)** Spearman rank correlations for the invCDw and the DAI (green line) and the SLN and the DAI (gray line, a reproduction of results by Mejias et al., 2016) as functions of frequency. The inset shows the p-values. The invCDw shows a significant negative correlation in the alpha-band and a significant positive correlation in the gamma-band similar to that found for the SLN. Interestingly, the invCDw shows almost the exact level of correlation in the alpha-band (for feedback connections) as the SLN, whereas in the gamma-band (for feedforward connections) it is somewhat lower. **(D)** There is a significant positive correlation between the invCDw and the multifrequency DAI (mDAI, *p* < 10^−6^).

The relationship between the topological, anatomical and functional hierarchies, namely CDw, SLN, and DAI was studied on the 8 × 8 subnetwork that was also analyzed by Mejias et al. (2016) (Figure 6A). Previous studies have shown a close relationship between SLN and DAI (Bastos et al., 2015), a fact that Mejias et al. (2016) built their model upon. Here we asked if CDw is correlated with SLN and especially with DAI. As Négyessy et al. (2008) reported, there is an inverse relation between CD and the hierarchical characteristics of the connections: positive CD values represent convergent edges, which in turn correspond to feedback communication, and an SLN value close to zero; conversely, negative CD edges are divergent and match feedforward connections, i.e., SLN values close to one. Accordingly, to make the comparisons more evident, the additive inverse of the CD (denoted as invCD) was used in the analysis. This did not change the results in any way, it only serves the purpose of a more consistent visualization.

**Figure 7:**
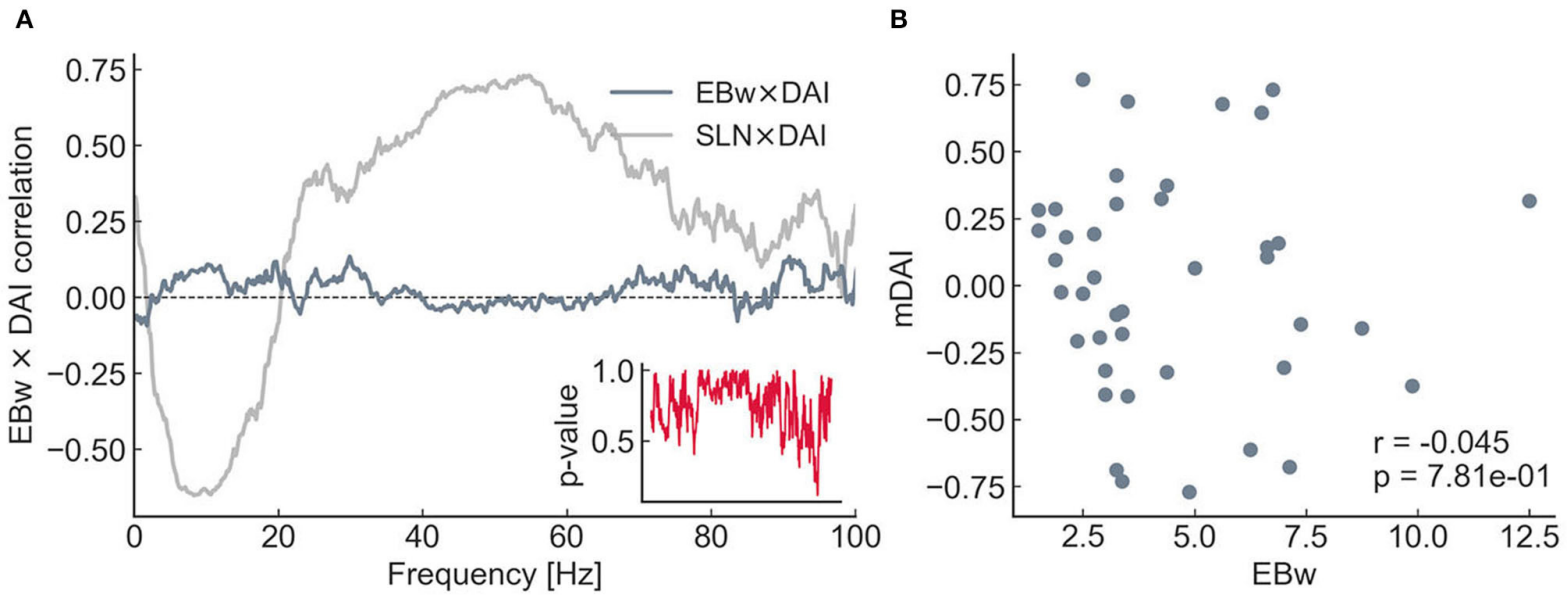
Relationship between the DAI and the edge betweenness (EBw with α and *k*) for the 8 × 8 subgraph. The layout is the same as for Figures 6C,D. In contrast with the CD, no significant correlations can be seen either with the DAI **(A)** or with the mDAI **(B)**.

It is important to emphasize that the invCDw was derived from edge properties that did not include any anatomical (SLN) or functional (DAI) hierarchical information, only the anatomical strength (the fraction of labeled projection neurons, FLN) and length (physical length of the projection in millimeters) of the connections used for the “relaxed” shortest path analysis. Therefore, it is quite remarkable, that a significant positive correlation was found between invCDw and SLN (*r* = 0.643, *p* < 1*e* − 5, Figure 6B), as well as between invCDw and DAI/mDAI (*r* = 0.668, *p* < 1*e* − 6 for mDAI, Figures 6C,D). Note that both comparisons were computed also with the binary CD of the graph, but the correlations were weaker in this case (*r* = 0.554, *p* < 1*e* − 3 with the SLN and *r* = 0.497, *p* < 1*e* − 3 with the mDAI, not shown), which is an indirect proof of the reliability of the relaxed weighted shortest path structure. Note also that in Figure 6C the frequency-dependent correlation between SLN and DAI (see SLN × DAI, gray line) was reproduced from Mejias et al. (2016). A remarkable finding was the close correspondence of the invCDw- and SLN-based correlations shown in Figure 6C, which was further studied as described in the following section. In contrast, the edge betweenness of the relaxed weighted shortest path structure did not show any of these correlations (Figure 7).

**Figure 8:**
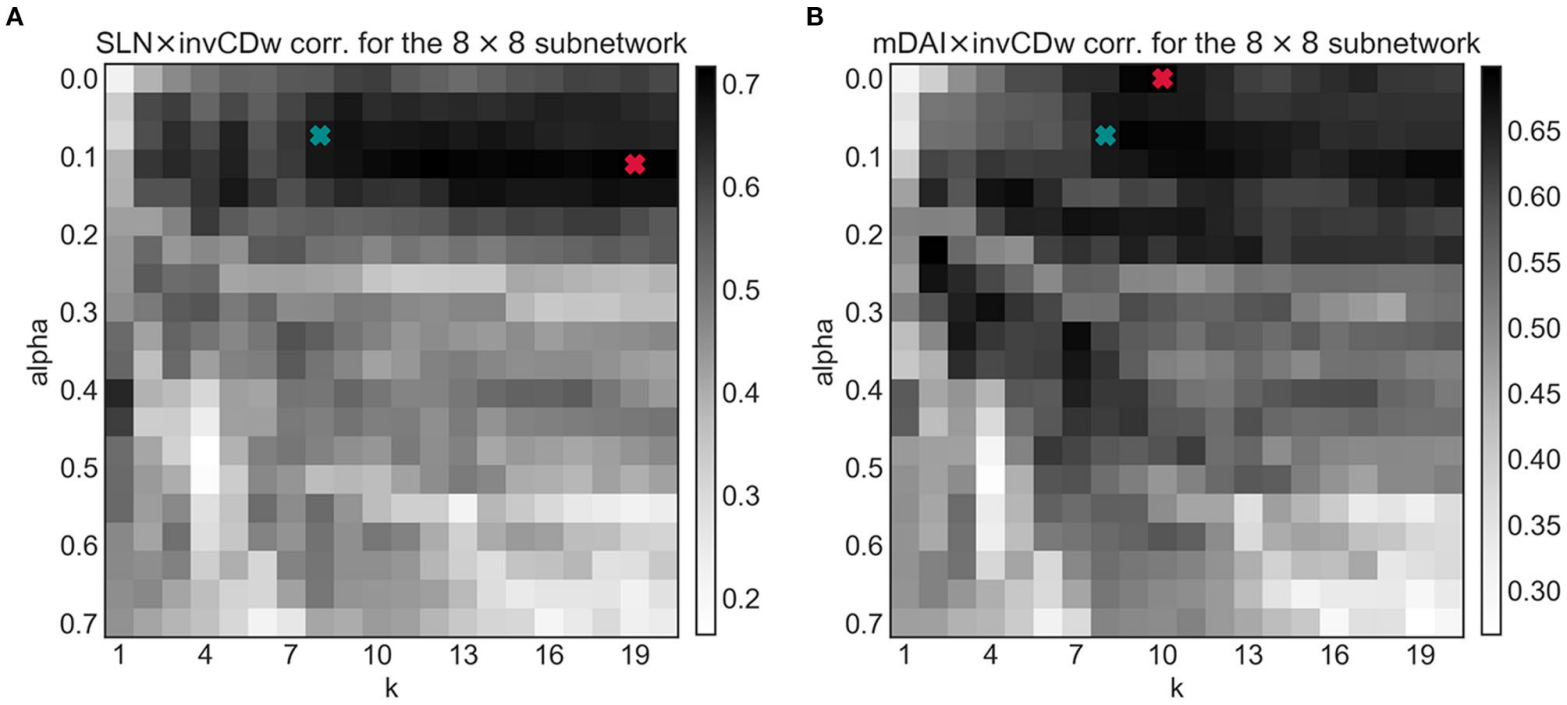
The tuning parameter dependencies of the linear correlations for the invCDw with the SLN **(A)** and with the mDAI **(B)** for the 8 × 8 subnetwork. Blue markers show the optimized α and *k* (see Figure 4), while red markers denote the absolute maxima of the 2D arrays. Note that the blue markers fall in the highly optimal regime in both cases. The notations are the same as in the previous figures.

Since the shortest path structure of the weighted graph has two arbitrary parameters it was important to see how these affect the observed correlations. To test this, the joint optimization of the α and *k* parameters was computed (Figure 8). In general, for both the SLN- and the mDAI-correlation increasing α (i.e., assigning greater importance to the weights and less to the number of jumps) decreases the magnitude of the correlation. After setting α to the optimal range ca. between 0.25 and 0.75 increasing *k* also increased the correlations in both cases.

According to Figure 8A, to achieve a maximal correlation in the invCDw × SLN case, an α of 0.1 and a very high value for *k* (specifically 19 by limiting its maximal value to 20) would fit the most; in the EB-based tuning procedure for *k* (Figure 4B) such high values were not even considered (only *k* ∈ [1, 15]). Notably, the correlations of CDw with SLN and mDAI differed regarding the most optimal values of α and *k*. According to Figure 8B, the correlation between invCDw and mDAI shows a maximum for the parameter values α = 0 and *k* = 10, but there are several other combinations that are only slightly lower. Although the results of our EB-based optimization (α = 0.07 and *k* = 8) were slightly less optimal for both cases, it can be surmised that using the higher values determined by invCDw × SLN and invCDw × mDAI would mean overfitting the data. Therefore, the EB-optimized values provide a more general solution, showing indirect support for the usefulness of our relaxed weighted shortest path detection technique. Further testing might illuminate the exact nature of the dependency of the correlations on α and *k*.

#### 3.2.4. Correlations in Samples of 8 × 8 Subgraphs

**Figure 9:**
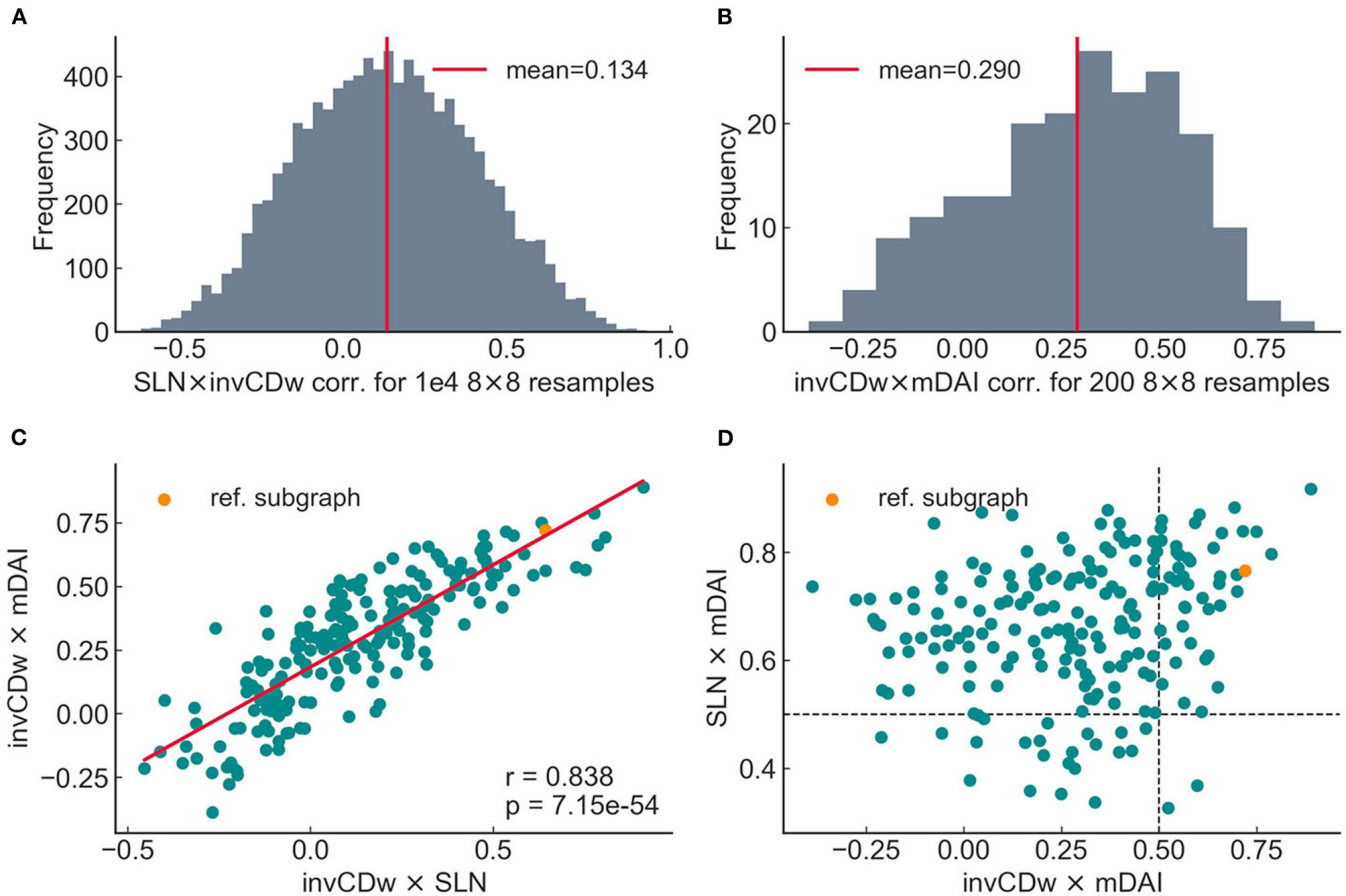
Relationship between SLN, DAI, and invCDw in 8 × 8 subgraphs sampled randomly from the entire 29 × 29 graph. **(A)** Distribution of the SLN × invCDw correlation values in 10,000 unique 8 × 8 subgraphs. **(B)** Distribution of the invCDw × mDAI correlation values in 200 unique 8 × 8 subgraphs. The relatively small number of samples is due to the simulation of the dynamics (and therefore the DAI) being very expensive computationally. **(C)** A strong correlation was found between the invCDw × SLN and the invCDw × mDAI correlations in the 200 random samples. **(D)** In general, invCDw × mDAI and the SLN × mDAI did not correlate in the 200 random samples. Strong correlation appeared only in a relatively small number of higher values (upper right quadrant demarcated by the dashed lines). The orange dot indicates the reference 8 × 8 subgraph studied with higher detail. The notations are the same as in the previous figures.

To unravel the correlations found on the previously selected 8 × 8 subgraph, the simulations were repeated on random samples of subgraphs with matching size taken from the entire visual cortical network. Reducing the size of the network to 8 nodes was also justified by the necessity to keep computational requirements below the capacity limit since the inclusion of more nodes in the dynamical simulations and especially the calculation of causality is computationally highly demanding tasks; the computation of the anatomical and topological correlations did not require such extra computational capacities. The correlation of SLN and invCDw was therefore computed in 10,000 (Figure 9A), while mDAI correlations were computed only for 200 unique random samples (Figure 9B). In both cases the histograms show that the correlation coefficients vary in a wide range ([−0.619, 0.928] for SLN and [−0.387, 0.889] for mDAI), and that the reference 8 × 8 subgraph resides in the positive tail of the distributions (*r* = 0.643 with SLN and *r* = 0.668 with mDAI); although there were subgraphs in which the correlations were even higher.

The joint distribution of invCDw × SLN and invCDw × mDAI values exhibited a highly significant positive correlation (Figure 9C) implying that the invCDw correlates with mDAI through the SLN. The reason for this strong correlation is that SLN is includedas a parameter in the dynamical model (Mejias et al., 2016; see also Supplementary Table 1). However, it is also clear that high correlations exist only for a small set of the randomly selected 8 × 8 sub-networks (Figure 9C). The joint distribution of the invCDw × mDAI and the SLN × mDAI values (Figure 9D) supported this observation by exhibiting a stronger relationship toward higher values. However, there was also a relatively large variability, especially for lower values.

**Figure 10:**
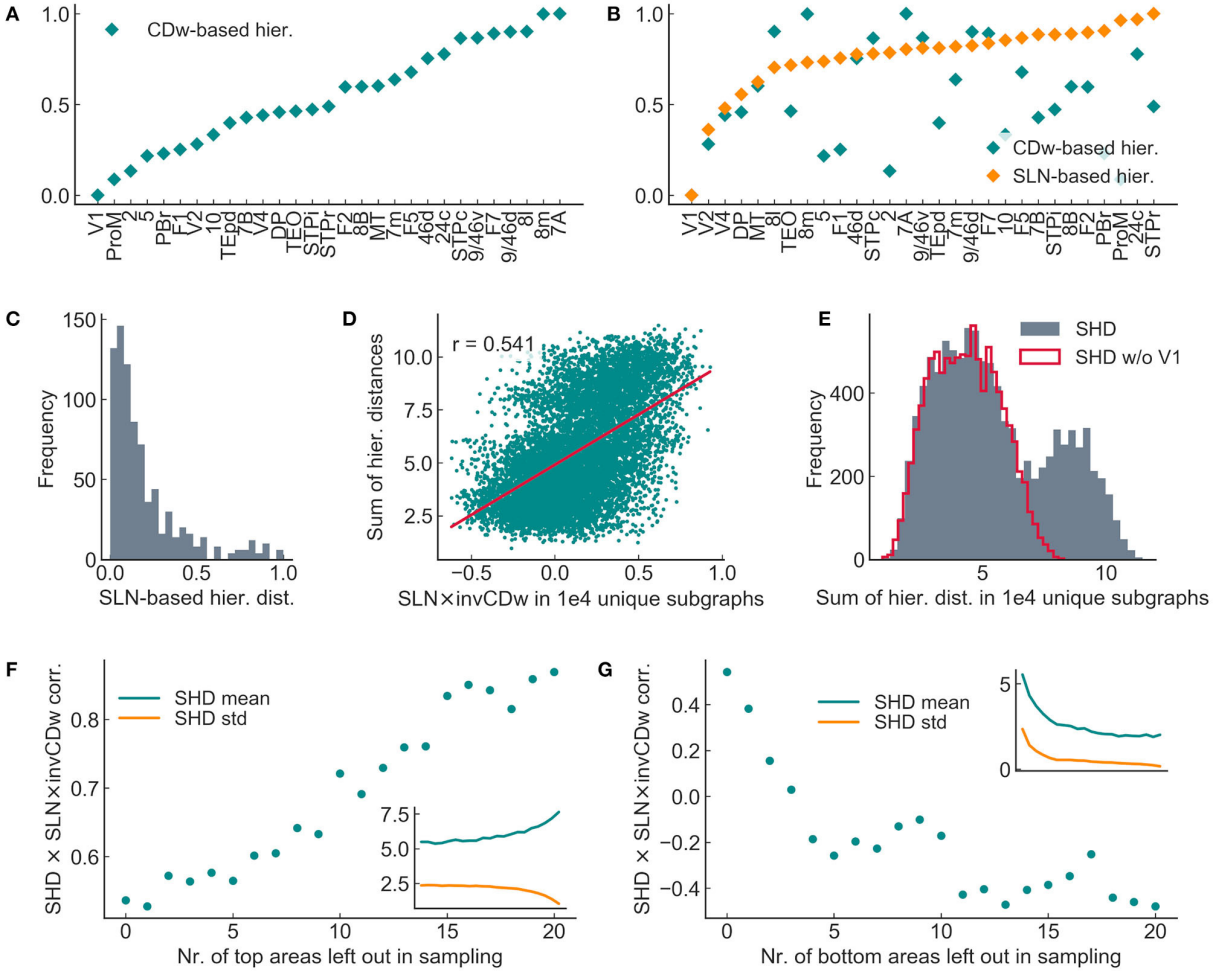
Spread of hierarchical distance determined the SLN × invCDw correlation in 8 × 8 subgraphs sampled randomly from the entire 29 × 29 graph. **(A)** CD-based hierarchy of areas in the entire graph. **(B)** SLN-based hierarchy of areas in the entire graph, with the corresponding CD-based hierarchical values also shown. The two hierarchies did not correlate in the full graph. **(C)** Distribution of SLN-based hierarchical distances for the entire graph. **(D)** The sum of SLN-based hierarchical distances (SHD) showed a highly significant positive correlation with the SLN × invCDw correlation in the random sample of subgraphs. **(E)** The overall large hierarchical distances of V1 (see **B**) significantly influenced the distribution of sample sums of hierarchical distances (SHDs). The bimodal histogram turned to unimodal after omitting V1 (SHD w/o V1). **(F,G)** The SHD and SLN × invCDw correlation changed in a consistent manner by leaving out nodes from subgraph sampling according to their position in the SLN-based hierarchy. The omission of the top areas increased the correlation **(F)** while leaving out bottom areas decreased the correlation **(G)**. This result implies that in the ideal case of large anatomical hierarchical distances, the correlation between the invCDw and the SLN and thereby between the invCDw and the mDAI would be maximal. The notations are the same as in the previous figures.

Therefore we looked for other factors that might determine the correlation magnitudes, especially considering the CDw- and the SLN-based hierarchies. As shown in Figures 10A,B both hierarchies change gradually. However, the majority of the areas had a very different position in the two hierarchies, i.e., areas exhibited different anatomical and topological properties (Figure 10B). Furthermore, in the topological, CDw-based hierarchy the areas align mostly linearly, while in the anatomical, SLN-based hierarchy the hierarchical distances have a long-tailed distribution (Figure 10C). Exploratory analysis suggested that the SLN × invCDw correlation depends primarily on the sum of SLN-based hierarchical distances in the random subgraphs, i.e., the total sum of the given 8 × 8 adjacency matrix containing the absolute differences in the anatomical hierarchical values between each pair of areas (sum of hierarchical distances, SHD; SLN × invCDw corr. with SHD in 10,000 samples, *r* = 0.541; Figure 10D). The correlation was similar in the 200 samples, where dynamics and causality were computed (invCDw × mDAI corr. with SHD in 200 samples, *r* = 0.479, *p* < 1*e* − 12). This fact is mostly due to area V1, which acts almost as an outlier, making the distribution of the sample sum of hierarchical distances bimodal (Figure 10E). The hypothesis that SLN × invCDw correlation depends mostly on the magnitude of hierarchical distances is further corroborated by the finding that leaving out areas in the random sampling procedure according to their place in the SLN-based hierarchy changes the SLN × invCDw correlation in a predictable manner (Figures 10F,G). Specifically, if areas from the top of the hierarchy (where hierarchical distances are small) were left out, the SLN × invCDw correlation increases. In contrast, leaving out areas from the bottom of the hierarchy (where distances are large) decreased the SLN × invCDw correlation.

## 4. Discussion

Despite its clinical relevance (van den Heuvel and Fornito, 2014; Griffis et al., 2019; Horien et al., 2020), few studies investigated the effects of link failure on the structural and functional integrity of cortical networks. The targeted edge removal used in this study provides evidence indicating that distinct topological properties of links play different roles in the vulnerability and thereby the functionally important structural properties of the network of the cerebral cortex, which can have important clinical implications. Kaiser and Hilgetag (2004) and Kaiser (2007) have shown that the cortical network is particularly vulnerable to edge betweenness (EB)-based edge elimination. We found here that EB is more important in communication efficiency of the network by showing that EB-based attack to links affects mostly the average shortest path and diameter. On the other hand, convergence degree (CD) was found to play a significant role in synchronizability, i.e., the propensity for oscillatory dynamics of the cortical network. We also found that synchronizability was affected by the targeted elimination of the divergent forward connections and showed mild sensitivity when attacking the convergent backward connections, which point to the importance of hierarchical dynamics in brain diseases.

Up to now convergence degree has been computed only in binary networks (Négyessy et al., 2008, 2012; Bányai et al., 2011b). Our findings show that the CD can also be computed for weighted networks (denoted CDw), increasing the power of this index zin network analysis. To obtain a realistic representation of CDw, we introduced a new joint optimization technique of weights that results in finding an improved, *relaxed* weighted shortest path structure of the large-scale cortical network. The relaxed shortest path structure shows robustness by having several alternative pathways between every node pair (in contrast to the single one found via the standard Dijkstra's algorithm), and it also solves the problem of unrealistically high binary lengths that the purely weight-minimizing approach results in Opsahl et al. (2010) and Avena-Koenigsberger et al. (2018). Furthermore, unlike other measures, specifically SLN and EB, CDw exposed a densely connected component of higher-order areas resembling the rich club of the cortical network, further supporting the significance of CD in understanding the functionally and pathologically important topological properties of the cerebral cortex (Collin et al., 2016; Griffa and Van den Heuvel, 2018). Interestingly, a strong correlation was found between the CDw-based topological hierarchy and the experimentally observed SLN-based hierarchy in a subnetwork studied previously by experimental and modeling investigations (Bastos et al., 2015; Mejias et al., 2016). In contrast, EB neither correlated with CD nor with SLN. However, random sampling of subnetworks indicated that correlations between CD and SLN exist only in a small subpopulation of subnetworks in the visual cortex. The highly correlated population of subnetworks exhibited large aggregate hierarchical distances between the constituting areas. We showed that in accordance with the SLN-based anatomical hierarchy, where only a few bottom level areas exhibit high distances while at higher levels there is only a smooth gradual change, removing the bottom areas from the sampling results in a significant decrease of correlation between CDw and SLN. These observations provide further insight into the flexibility of hierarchical organization both in terms of topology and dynamics (Hilgetag et al., 2000; Mejias et al., 2016; Vezoli et al., 2021).

### 4.1. Complementary Role of Associational Connections in Network Organization and Cortical Pathology

The results of this study support previous observations on the vulnerability of the large-scale network of the cerebral cortex to edge removal and extend them by showing that different edge properties, based on network topology, play a complementary role in network integrity. The present analysis highlights the significance of CD in the vulnerability of the cortical network by showing that removing edges based on CD values affects characteristic network properties as much as found after EB-based removal of the connections (Kaiser and Hilgetag, 2004; Kaiser, 2007). Accordingly, the cortical network fragmented quickly due to targeted attacks, as measured by the number of strongly connected components. The cortex was resilient against random attack in terms of strong connectedness. Regarding aggregation, the network appeared to be robust against all kinds of edge removal strategies as shown by the slow and initially mild change in transitivity. In fact, transitivity was the most sensitive to random edge deletion, due probably to the relatively high level of reciprocity of the cortical network analyzed.

Global efficiency plays an important role in network communication (Avena-Koenigsberger et al., 2018). Measured by the average shortest path and diameter, the global efficiency was sensitive to targeted attacking strategies but robust to random edge removal similarly as reported previously (Kaiser and Hilgetag, 2004; Kaiser, 2007). We found that efficiency depends more on EB than CD. In contrast, synchronizability exhibited higher vulnerability to CD- than EB-based targeting. These findings about the complementary role of EB and CD in vulnerability can be explained by the fact that both are computed from shortest paths, therefore these are not mutually exclusive but rather complementary indices. Also, the fact that EB represents the number of shortest paths can explain why global efficiency relies more on EB than CD. On the other hand, by expressing the relative number of input and target areas connected by shortest paths, CD apparently captures a deeper meaning of network topology and makes it a closer counterpart of spectral graph metrics (Newman, 2003; Boccaletti et al., 2006).

An interesting observation of the present study was the different role of edges with divergent and convergent CD properties (characterizing forward and backward connections, respectively) in network resilience. While targeting divergent forward connections significantly affected synchronizability, elimination of convergent backward links exhibited small effects on network integrity. Considering that synchronizability is sensitive to bottleneck effects, our findings suggest that weakening forward interactions results in disintegration of the network by detaching source nodes. In contrast to algebraic connectivity, the largest eigenvalue is robust to link failure, which shows that the synchronizing capacity remains high even in a fragmented form of the network. These apparently contradictory findings can be resolved by considering the presence of a robust, densely connected rich club of high degree nodes, as shown previously and also in the present study (Harriger et al., 2012; Griffa and Van den Heuvel, 2018). Accordingly, in agreement with our findings that show the sensitivity of the largest eigenvalue to the heterogeneity of degree distribution (i.e., the higher sensitivity of the cortical and rewired networks compared to the ER random graph), the largest eigenvalue depends strongly on the degree of vertices and paths connecting them (Barahona and Pecora, 2002; Nishikawa et al., 2003; Arenas et al., 2008; Chen et al., 2012). Interestingly, edge removal changes the absCD, which suggests that forward-like and backward-like convergence properties of the links can change resulting in the reorganization of network topology. It follows that removing divergent edges not only detaches peripheral nodes or groups of them but turns the outputs of such nodes to highly divergent links connecting the few peripheral nodes to the relatively large core of the network. This view is consistent with the bow tie-like core-periphery organization of the hierarchical cortical network (Markov et al., 2013b). It is interesting to note, that the sensitivity of the largest eigenvalue to removing the convergent backward connections may indicate that the functionality of the rich club depends mostly on feedback interactions and may also point to the importance of top-down interactions in brain disease (Bassett et al., 2008; Bányai et al., 2011a; Silverstein et al., 2016; Raj and Powell, 2018; Perry et al., 2019). In addition, as edge removal changes the convergence properties of connections, it can result in the integration of inconsistent information and, as a consequence, may change the functional coupling of nodes in the core of the network. This hypothesis would be consistent with observations of weakening interactions between high degree nodes in brain disease (Bassett et al., 2018). Our findings make important contributions to the understanding of the network mechanisms of disconnection syndromes (Catani and Ffytche, 2005) or in a broader term connectopathy (Collin et al., 2016).

### 4.2. Partial Correlation of Topological and Anatomical Hierarchies Support Flexible Hierarchical Dynamics

The average correlation between SLN and CD is low, and due to the dynamical model used (which includes SLN as a coupling parameter), the CD has a low average correlation with mDAI representing the Granger causal hierarchical interactions between areas in the network analyzed. However, the CD can exhibit high correlation in subpopulations of interacting areas, but only in cases where SLN-based hierarchical distances between the areas are large. In the visual cortex, only low-level areas exhibit high SLN-based hierarchical distances. This observation suggests that CD-based topological hierarchy primarily shapes low-level visual cortical processing. However, the visual cortex is a subnetwork of the cerebral cortex, and the hierarchical ordering, especially the one based on CD, can change by including more areas. As long as the complete anatomical network of the macaque cerebral cortex is not available, the question of correlation between SLN- and CD-based hierarchies cannot be clarified.

Another potential difficulty regarding the correlation of anatomical and topological hierarchies is that both SLN and CD exhibits smooth gradual change. In the case of SLN, except for the few low-level areas at the bottom, the hierarchical distance is very small, especially between middle-tier areas in the visual cortex (Mejias et al., 2016; Vezoli et al., 2021). Therefore, slight changes can result in very different ordering in the hierarchical position. Accordingly, the hierarchy of the cerebral cortex is not completely determined (Hilgetag et al., 2000), even when computed by exact metrics like SLN (Vezoli et al., 2021). In fact, the cerebral cortex exhibits hierarchical organization in many different means including anatomical, physiological and topological (Hilgetag and Goulas, 2020). Therefore, the organizational complexity allows some functional flexibility via the interplay of these multiple kinds of hierarchical characteristics, which is supported both by experimental observations and modeling, showing the hierarchical jump of areas in response to changing patterns of activities (Bastos et al., 2015; Mejias et al., 2016).

Further hierarchical uncertainties and larger computational flexibility may arise from the dual counterstream organization of the feedforward (FF) and feedback (FB) pathways, which, in addition to the major stream of mid-upper layer FF and deep layer FB organization, opens possibilities for top-down and bottom-up interaction within the upper and deep layers of the cerebral cortex, not only across them (Markov et al., 2013a; Vezoli et al., 2021). However, this complexity of inter-areal hierarchical circuitry was not included in the model applied here, and it is hard to predict its exact role in spectral causal coupling in the cortex with diverse oscillatory dynamics (Buzsáki and Draguhn, 2004; Wang, 2010).

Altogether, the results of this study indicate that network topology based on CD fits well with hierarchical dynamics until the anatomical and topological hierarchies correlate strongly. However, the diverse ways of hierarchies may contribute differently to cortical dynamics in a contextual manner (Bastos et al., 2020; Vezoli et al., 2021). Accordingly, an architecture with multiple and gradually changing hierarchies provides room for a flexible, dynamical rearrangement of functional hierarchies (Bastos et al., 2015; Mejias et al., 2016; Hilgetag and Goulas, 2020). Most notably, this flexibility of interaction dynamics seems to be a characteristic feature of high-level areas forming the core of the cerebral cortex.

## Data Availability Statement

The original contributions presented in the study are included in the article/Supplementary Material, further inquiries can be directed to the corresponding authors.

## Author Contributions

LN and BV designed the study and wrote the manuscript. BS and BJ performed edge elimination experiments and analyzed data. BV performed dynamic network modeling, introduced the relaxed shortest path measure and analyzed data. ZS supervised the dynamic network modeling and computation of spectral Granger causality and revised the manuscript. EB updated the binary visuo-tactile network. All authors contributed to the article and approved the submitted version.

## Conflict of Interest

The authors declare that the research was conducted in the absence of any commercial or financial relationships that could be construed as a potential conflict of interest.

## Footnotes

^1^http://core-nets.org/

^2^http://igraph.org/r/

^3^https://senselab.med.yale.edu/ModelDB/showmodel.cshtml?model=249589

^4^https://github.com/Eden-Kramer-Lab/spectral_connectivity
