# Supplementary material for: Network Path Convergence Shapes Low-Level Processing in the Visual Cortex
Source: Front Syst Neurosci. 2021 May 24;15:645709. doi: 10.3389/fnsys.2021.645709 (PMC8181740; doi:10.3389/fnsys.2021.645709)
Supplement: Supplementary file 3 [file Data_Sheet_1.PDF]

Supplementary Methods

1 DYNAMICAL MODEL

The multi-scale model describes four embedded levels of neuronal structure: i) local excitatory and inhibitory populations in a given cortical layer, ii) four populations, two for the supragranular and two for the infragranular layer, representing a macroscopic cortical area, iii) two coupled areas, both represented by a laminar model, and iv) the large-scale cortical network of 29 areas, mainly in the visual pathways. The model was published in Mejias et al. (2016); the following is a reiteration of their work.

1.1 Intralaminar local circuit

The basic building block of the model is a pair of neuronal populations, excitatory and inhibitory, representing the respective pyramidal cells and interneurons in one (supra- or infragranular) layer of a given cortical region. Mathematically, a nonlinear Wilson–Cowan-type neuronal mass model is employed, described by a pair of coupled stochastic differential equations of the forms:

$$\tau_E \frac{dr_E}{dt} = -r_E + \Theta(I_E^{net} + I_E^{ext}) + \sqrt{\tau_E}\xi_E(t), \tag{S1}$$

$$\tau_I \frac{dr_I}{dt} = -r_I + \Theta(I_I^{net} + I_I^{ext}) + \sqrt{\tau_I}\xi_I(t), \tag{S2}$$

where $r_{E,I}$ are dimensionless activities (i.e. mean firing rates) of the excitatory and inhibitory populations, respectively, $\tau_{E,I}$ are the corresponding time constants, $\xi_{E,I}$ are Gaussian noise terms with zero mean, and $\sigma_{E,I}$ strength and $\Theta(x) = x/(1 - \exp^{-x})$ is the transduction function (used instead of a sigmoid function), while $I_{E,I}^{net}$ and $I_{E,I}^{ext}$ represent the input from other parts of the network and from exterior sources (e.g from the thalamus, which is not included as an individual node), respectively. If we assume isolated intralaminar populations with only local contributions, then the network input is:

$$I_E^{net} = J_{EE}r_E + J_{EI}r_I, \tag{S3}$$

$$I_I^{net} = J_{IE}r_E + J_{II}r_I, \tag{S4}$$

where $J_{\alpha\beta}$ is the coupling strength (mean synaptic strength), from population β to population α. The populations are connected both reciprocally and recurrently. Note that in the absence of all inputs (recurrent, network and external) to a population the equations still produce oscillations, but if we cut the noise term too, the mean activities die down exponentially. Two different parameter sets are employed in accordance with electrophysiological studies (Bastos et al., 2015; van Kerkoerle et al., 2014), one for the supragranular and one for the infragranular populations: the former is chosen to achieve a noise-driven gamma (40 Hz) rhythm, while the latter shows alpha to lower beta (10-30 Hz) oscillations; see Table S1 for the exact parameter values used in this study. A case of elevated activity can be simulated by a constant external input to the excitatory populations (e.g. the effect of contrast in V1).

1.2 Interlaminar local circuit

The next level of the model couples the supra- and infragranular layers (L2 and L5 in the mathematical notation, for clarity) to form a local circuit, which is assumed to represent an entire cortical area. There are 2×2 populations, thus four different potential projections, of which only the two strongest are used, according to the anatomical studies (Markov et al., 2014): one from the supragranular excitatory (L2E) to the infragranular excitatory (L5E) population and one from the infragranular excitatory (L5E) to the supragranular inhibitory (L2I) population. This is achieved by extending equations S3 and S4. Using a convenient matrix notation, the input arriving to each of the four populations is

$$\begin{bmatrix} I_{L2E} \\ I_{L2I} \\ I_{L5E} \\ I_{L5I} \end{bmatrix} = \begin{bmatrix} J_{EE} & J_{EI} & 0 & 0 \\ J_{IE} & J_{II} & J_{2,5} & 0 \\ J_{5,2} & 0 & J_{EE} & J_{EI} \\ 0 & 0 & J_{IE} & J_{II} \end{bmatrix} \begin{bmatrix} r_{L2E} \\ r_{L2I} \\ r_{L5E} \\ r_{L5I} \end{bmatrix}, \tag{S5}$$

with $J_{2,5}$ and $J_{5,2}$ being the strength of the interlaminar projection from supra- to infragranular and infra- to supragranular layer, respectively. For a given area α we can rewrite this in a compact notation as $\mathbf{I}_\alpha = \mathbf{J}_\alpha \mathbf{r}_\alpha$. The result is what we would expect: via these interlaminar projections intralaminar rhythms can spread across layers and modulate each other's intrinsic oscillations.

1.3 Interareal model

Next, two of the previously described interlaminar circuits are coupled, simulating the interaction of two distinct cortical areas. Thus far, the model does not differentiate between areas: the connectivity pattern of the local circuits, as well as their parametrization is assumed to be universal across the cortex. The layer-specific connection strengths between the individual areas however depend on their relative positions in the anatomical hierarchy (Felleman and Van Essen, 1991; Markov et al., 2014).

The area pair of V1 and V4 have a distinctive hierarchical relationship, so that the projection from V1 to V4 is exclusively feedforward (FF), while from V4 to V1 it is solely feedback (FB, note that in the large-scale case a more general approach will be used, where projections are mixtures of FF and FB communication, according to their SLN value, i.e. the fraction of supragranular labelled neurons; see the main text for further description). Anatomical studies show that in the visual pathway feedforward projections originate mainly from L2/3 pyramidal neurons and target layer 4 excitatory neurons, which in turn establish synaptic connections with L2/3 pyramidal neurons (Markov et al., 2014; Mejias et al., 2016); this is approximated with an interareal projection from L2/3E of the source area to L2/3E of the target area. Feedback communication in turn mainly originates in L5/6E but is not so focused, giving collaterals to all four populations of the supra- and infragranular layer. This is represented with two distinct input equations for the two areas:

$$\mathbf{I}_{V4} = \begin{bmatrix} I_{V4L2E} \\ I_{V4L2I} \\ I_{V4L5E} \\ I_{V4L5I} \end{bmatrix} = \mathbf{J}_{V4}\mathbf{r}_{V4} + \begin{bmatrix} J_{FF1} & 0 & 0 & 0 \\ 0 & 0 & 0 & 0 \\ 0 & 0 & 0 & 0 \\ 0 & 0 & 0 & 0 \end{bmatrix} \begin{bmatrix} r_{V1L2E} \\ r_{V1L2I} \\ r_{V1L5E} \\ r_{V1L5I} \end{bmatrix}, \tag{S6}$$

for the feedforward direction, which can be written as $\mathbf{I}_{V4} = \mathbf{J}_{V4}\mathbf{r}_{V4} + \mathbf{J}_{FF}\mathbf{r}_{V1}$, and

$$\mathbf{I}_{V1} = \begin{bmatrix} I_{V1L2E} \\ I_{V1L2I} \\ I_{V1L5E} \\ I_{V1L5I} \end{bmatrix} = \mathbf{J}_{V1}\mathbf{r}_{V1} + \begin{bmatrix} 0 & 0 & J_{FB1} & 0 \\ 0 & 0 & J_{FB2} & 0 \\ 0 & 0 & J_{FB3} & 0 \\ 0 & 0 & J_{FB4} & 0 \end{bmatrix} \begin{bmatrix} r_{V4L2E} \\ r_{V4L2I} \\ r_{V4L5E} \\ r_{V4L5I} \end{bmatrix}, \tag{S7}$$

for the feedback stream, also compacted to $\mathbf{I}_{V1} = \mathbf{J}_{V1}\mathbf{r}_{V1} + \mathbf{J}_{FB}\mathbf{r}_{V4}$.

To simulate an electrophysiological measurement, the weighted combination of the population activity is calculated for each area, as:

$$S_\alpha(t) = (1-\eta)r_{\alpha L2E}(t) + \eta r_{\alpha L5E}(t) \tag{S8}$$

representing the signal measured by an electrode recording in area α, with the parameter η reflecting the depth in the cortex. The two areal signals show a frequency-specific relationship in each direction; to resolve the directionality of influences in the joint spectrum, the multivariate spectral Granger causality (GC) analysis and directed influence asymmetry index (DAI, see the main text) is employed. This reveals the two distinct peaks for the FF and FB communication, in the gamma and alpha band, respectively, supporting electrophysiological observations Bastos et al. (2015); van Kerkoerle et al. (2014).

1.4 Large-scale model

To construct the large-scale network model, the anatomical connectivity data and indices described by Markov et al. (2014) are used. The graph has 29 vertices, with a density of 66%. A multilaminar circuit is set in each node, and the edge weights are defined as follows. (i) The FLN value (fraction of labelled neurons; see the main text for further description) of the corresponding anatomical projection serves as a basic strength for the edge, after a log-linear transformation, addressing the fact that FLN values span a range of about five orders of magnitude. Using $w_{ij} = c_1 FLN_{ij}^{c_2}$ with optimal parameter values for c_1 and c_2, the connectivity information is preserved, while the actual strength values are compressed to a range that the rate model can handle effectively. (ii) The SLN value of the projection characterizes the hierarchical relationship between the areas, and therefore the laminar specificity of the origin and target of the projections. An SLN value of unity would mean that the communication on the edge is totally FF, while a zero SLN signifies a pure FB direction; an SLN in between represents a specific proportion of the two, with a factor of SLN_{ij} for the FF and $(1 - SLN_{ij})$ for the FB flow. (iii) Lastly, while at the local level the closeness of the populations did not warrant a delay in transmission, on the large-scale level a mean empiric delay term is included, calculated as the product of the reported anatomical length of the projections and an average action potential propagation speed of 1.5 m/s.

Putting together all these components, the net input received by neuronal populations in a given area i at time t is

$$\mathbf{I}_i(t) = \mathbf{J}_i\mathbf{r}_i(t) + \sum_{j\neq i}^{areas} \left[W_{FF}^{ij}\,\mathbf{J}_{FF} + W_{FB}^{ij}\,\mathbf{J}_{FB}\right]\mathbf{r}_j(t-\Delta_{ij}), \tag{S9}$$

where $W_{FF}^{ij} = w_{ij}SLN_{ij}$ and $W_{FB}^{ij} = w_{ij}(1-SLN_{ij})$ are the total weights for the FF and FB component, respectively (with w_{ij} as defined above), and Δ_{ij} is the interareal delay term. The network also receives a

uniform constant external ('thalamic') input to every area, plus a constant external input on top of this to area V1, representing a strong stimulation arriving from the retina.

In this final form of the model, while the time series data was simulated for the entire 29-node network, following the example of Mejias et al. (2016) and Bastos et al. (2015), due to computational limitations, we analyzed primarily a subnetwork of 8 regions of interest in terms of causal interactions. These 8 regions were selected roughly evenly from the visual hierarchy and are namely: V1, V2, V4, DP, 8m, 8l, TEO and 7A. Lastly, to verify the results found on this special subnetwork, additional simulations were run on a batch of randomly sampled 8×8 subnetworks, too.

1.5 Notes on simulation

The model is essentially a large system of coupled stochastic differential equations, which we solved using the Euler–Maruyama method. The simulations were run with a dt value of $0.2\,ms$, corresponding to a sampling frequency of $5\,kHz$. For most cases of laminar simulations, a total time period of $T = 30\,s$ was generated, with a transient of $5\,s$. For the interareal and large-scale model, much longer time periods are needed, specifically $T = 180\ s$ with a transient of $20\ s$ in our study. The causality estimations were done on downsampled time series, taking the mean of every 25 time points; the same smoothing measure also had to be applied to the causality estimations. The averaging and smoothing served only the purpose of noise reduction and did not distort the data in any significant way.

Data for the 29×29 network were collected from the publicly available core-nets database (`http://core-nets.org/`). Relevant information from tables `PNAS_2013.xlsx`, `Neuron_2015_Table.xlsx` and `PLOS_Biol_2016.zip` were edited together and included as table `Weighted_Visual_29x29.xlsx` of the Supplementary Material.

2 SLN-BASED HIERARCHY

In reconstructing the SLN-based hierarchy we referred to the methods of Markov et al. (2014), who applied a general linear model to their SLN data. A beta-binomial model is fitted by maximum likelihood, using a logit link (Markov et al. (2014) used a probit link, but this did not make a significant difference (Markov et al., 2014)). For fitting the *betabin* function was used from the aod R package with the code:

```
betabin( cbind(Supra, Infra) ~ X[,-1] + 0,
         random = ~ 1, link = logit, data = DTA ),
```

where `DTA` is a dataframe, containing all edges as rows and four columns: the name of the source area (`Source`), the name of the target area (`Target`), the number of supragranular projecting neurons (`Supra`) and the number of infragranular projecting neurons (`Infra`). The data frame `X` is the incidence matrix of the graph, containing a row for each edge, a column for each area, and the cell value -1, +1 or 0, where the source area is -1, the target +1, and the rest is 0. The `[,-1]` indexing omits the first column of `X` (area V1), which is assumed to be the base of the hierarchy (having a 0 value). The `random` term provides an estimate of the dispersion in the model fit.

Symbol	Value	Description
c_1	1.2	FLN log-linear transformation param. 1
c_2	0.3	FLN log-linear transformation param. 2
η	0.8	simulation electrode depth param.
I_E^{ext}	6 (12 for V1 supragr.)	external constant (thalamic) input to exc. pop.
I_I^{ext}	0	external constant (thalamic) input to inh. pop.
J_{EE}	1.5	exc. to exc. intralaminar conn. str.
J_{EI}	–3.25	inh. to exc. intralaminar conn. str.
J_{IE}	3.5	exc. to inh. intralaminar conn. str.
J_{II}	–2.5	inh. to inh. intralaminar conn. str.
$J_{5,2}$	1	supragr. to infragr. interlaminar conn. str.
$J_{2,5}$	0.75	infragr. to supragr. interlaminar conn. str.
J_{FF1}	1	supragr. to supragr. FF interareal conn. str.
J_{FB1}	0.1	infragr. inh. to supragr. exc. FB interareal conn. str.
J_{FB2}	0.5	infragr. inh. to supragr. inh. FB interareal conn. str.
J_{FB3}	0.9	infragr. inh. to infragr. exc. FB interareal conn. str.
J_{FB4}	0.5	infragr. inh. to infragr. inh. FB interareal conn. str.
σ_{L2}	0.3	standard deviation for supragr. noise term
σ_{L5}	0.45	standard deviation for infragr. noise term
τ_{L2E}	6 ms	time constant for supragr. exc. population
τ_{L2I}	15 ms	time constant for supragr. inh. population
τ_{L5E}	30 ms	time constant for infragr. exc. population
τ_{L5I}	75 ms	time constant for infragr. inh. population

Table S1. Parameter table. Abbreviations: exc. = excitatory, inh. = inhibitory, supragr. = supragranular, infragr. = infragranular, FF = feedforward, FB = feedback, pop. = population, conn. str. = connection strength.

REFERENCES

Bastos, A. M., Vezoli, J., Bosman, C. A., Schoffelen, J.-M., Oostenveld, R., Dowdall, J. R., et al. (2015). Visual areas exert feedforward and feedback influences through distinct frequency channels. *Neuron* 85, 390–401. doi:10.1016/j.neuron.2014.12.018

Felleman, D. J. and Van Essen, D. C. (1991). Distributed hierarchical processing in the primate cerebral cortex. *Cerebral Cortex* 1, 1–47. doi:10.1093/cercor/1.1.1

Markov, N. T., Vezoli, J., Chameau, P., Falchier, A., Quilodran, R., Huissoud, C., et al. (2014). Anatomy of hierarchy: Feedforward and feedback pathways in macaque visual cortex. *Journal of Comparative Neurology* 522, 225–259. doi:10.1002/cne.23458

Mejias, J. F., Murray, J. D., Kennedy, H., and Wang, X.-J. (2016). Feedforward and feedback frequency-dependent interactions in a large-scale laminar network of the primate cortex. *Science Advances* 2.

doi:10.1126/sciadv.1601335

van Kerkoerle, T., Self, M. W., Dagnino, B., Gariel-Mathis, M.-A., Poort, J., van der Togt, C., et al. (2014). Alpha and gamma oscillations characterize feedback and feedforward processing in monkey visual cortex. *Proceedings of the National Academy of Sciences* 111, 14332–14341. doi:10.1073/pnas.1402773111
